# Past connections with the mainland structure patterns of insular species richness in a continental‐shelf archipelago (Aegean Sea, Greece)

**DOI:** 10.1002/ece3.7438

**Published:** 2021-03-29

**Authors:** Cyril Hammoud, Konstantinos Kougioumoutzis, Kenneth F. Rijsdijk, Stylianos M. Simaiakis, Sietze J. Norder, Johannes Foufopoulos, Elisavet Georgopoulou, Emiel E. Van Loon

**Affiliations:** ^1^ Invertebrate Unit Department of Biology Royal Museum for Central Africa Tervuren Belgium; ^2^ Limnology Unit Department of Biology Ghent University Ghent Belgium; ^3^ Department of Biology Section of Ecology and Taxonomy National & Kapodistrian University of Athens Athens Greece; ^4^ Institute for Biodiversity and Ecosystem Dynamics University of Amsterdam Amsterdam The Netherlands; ^5^ Natural History Museum of Crete University of Crete Crete Greece; ^6^ Leiden University Centre for Linguistics Leiden University Leiden The Netherlands; ^7^ School of Natural Resources and Environment University of Michigan Ann Arbor MI USA

**Keywords:** Aegean archipelago, angiosperms, biogeography, butterflies, centipedes, land‐bridge island, last glacial maximum, Pleistocene, reptiles

## Abstract

Recent research in island biogeography has highlighted the important role of late Quaternary sea‐level fluctuations in shaping biogeographic patterns in insular systems but focused on oceanic systems. Through this study, we aim investigate how late Quaternary sea‐level fluctuations shaped species richness patterns in continental‐shelf island systems. Focusing on the Aegean archipelago, we first compiled maps of the area's geography using published data, under three sea‐level stands: (a) current; (b) median sea‐level over the last nine glacial–interglacial cycles (MSL); and (c) Last Glacial Maximum (LGM). We gathered taxon–island occurrences for multiple chorotypes of angiosperms, butterflies, centipedes, and reptiles. We investigated the impact of present‐day and past geographic settings on chorological groups by analyzing island species–area relationships (ISARs) and using generalized linear mixed models (GLMMs) selection based on multiple metrics of goodness of fit. Our results confirm that the Aegean's geography has changed dramatically since the LGM, whereas the MSL only modestly differs from the present configuration. Apart for centipedes, paleogeographic changes affected both native and endemic species diversity through altering connections between land‐bridge islands and the mainland. On land‐bridge islands, we detected over‐representation of native species and under‐representation of endemics. Unlike oceanic islands, sea‐level‐driven increase of isolation and area contraction did not strongly shape patterns of species richness. Furthermore, the LGM configurations rather than the MSL configuration shaped patterns of endemic species richness. This suggests that even short episodes of increased connectivity with continental populations are sufficient to counteract the genetic differentiation of insular populations. On the other hand, the over‐representation of native nonendemic species on land‐bridge islands reflected MSL rather than LGM mainland connections. Our study shows that in terms of processes affecting species richness patterns, continental archipelagos differ fundamentally from oceanic systems because episodic connections with the mainland have profound effects on the biota of land‐bridge islands.

## Graphical abstract

In the Aegean archipelago, the shorter lasting configurations of the Late Glacial Maximum promoted increased connectivity with the mainland, thereby counteracting processes promoting endemism. This result contrasts strongly with observations made on oceanic island, where longer lasting configurations largely shaped the current species diversity. This highlights that, in terms of processes affecting species richness patterns, continental archipelagos differ fundamentally from oceanic systems.

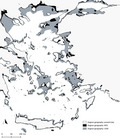

## 1 INTRODUCTION

Islands are dynamic entities with continuously evolving geographic settings influencing the distribution and evolution of organisms they host. At deep time scales (Ma), islands’ ontogeny shapes patterns of species diversity (Whittaker et al., 2008), whereas on shorter time scales’ climatic processes dominate (Fernández‐Palacios et al., 2015). Climate drives eustatic sea‐level fluctuations causing islands to shrink and expand, fragment and merge, or even disappear and emerge (e.g., Simaiakis et al., 2017). Late Quaternary sea‐level changes have left their imprint on insular species diversity patterns (Ali & Aitchison, 2014; Ávila et al., 2018, 2019; Norder et al., 2018, 2019; Rijsdijk et al., 2014; Weigelt et al., 2016). Most work, however, has focused on volcanic oceanic islands or did not make a distinction between oceanic and continental insular systems and lumped them in their analyses (Veron et al., 2019; Weigelt et al., 2016). By disregarding the specifics of continental archipelagos, relevant biogeographic processes are potentially overlooked (Ali, 2017; Simaiakis et al., 2017; Whittaker et al., 2017).

Continental‐shelf islands (sensu Ali, 2017) are characterized by sitting on a continental‐shelf and may include “land‐bridge” islands that were connected to the mainland in the past when sea levels were lower (Box 1). Another characteristic is the proximity to the mainland often surrounding continental islands (Weigelt & Kreft, 2013). The geo‐spatial effects of sea‐level dynamics on continental island biota have been studied extensively (e.g., Cardillo et al., 2008; Diamond, 1972; Itescu et al., 2020).

BOX 1 **A land‐bridge island is an island that was connected to a nearby mainland during an episode of lower sea level (e.g., the Last Glacial Maximum). Island formation occurs with the drowning of the land‐bridge connecting the landmass to the mainland. The timing of separation from the mainland may differ among land‐bridge islands of the same archipelago, depending on the topography of the peninsula and local heterogeneities in the dynamics of sea‐level rise. With continuing sea‐level rise, the isolation of the island is followed by a progressive decline of its area. Because insular area is a major determinant of species richness, variations in the magnitudes and rates of area loss are expected to drive varying rates of species loss. Here, we considered 3 hypothetical islands with the same initial area and undergoing the change of sea level to illustrate how the magnitude of species richness loss might differ depending on an island's topography (Box 1.A: I, II and III). Area loss of the first hypothetical island is gentle (Box 1.B: I), initially slow then fast for the second island (Box 1.B: II), and fast then slow for the third one (Box 1.B: III.). Species richness loss follows a similar trend though it occurs in a delayed fashion after area loss (Box 1.C: S0 to SP). Due to its larger remaining area, island I now hosts a larger richness of species than island II and III. Furthermore, species richness is expected to be at equilibrium in island I and III but not island II, as it lost a large share of its area recently.**

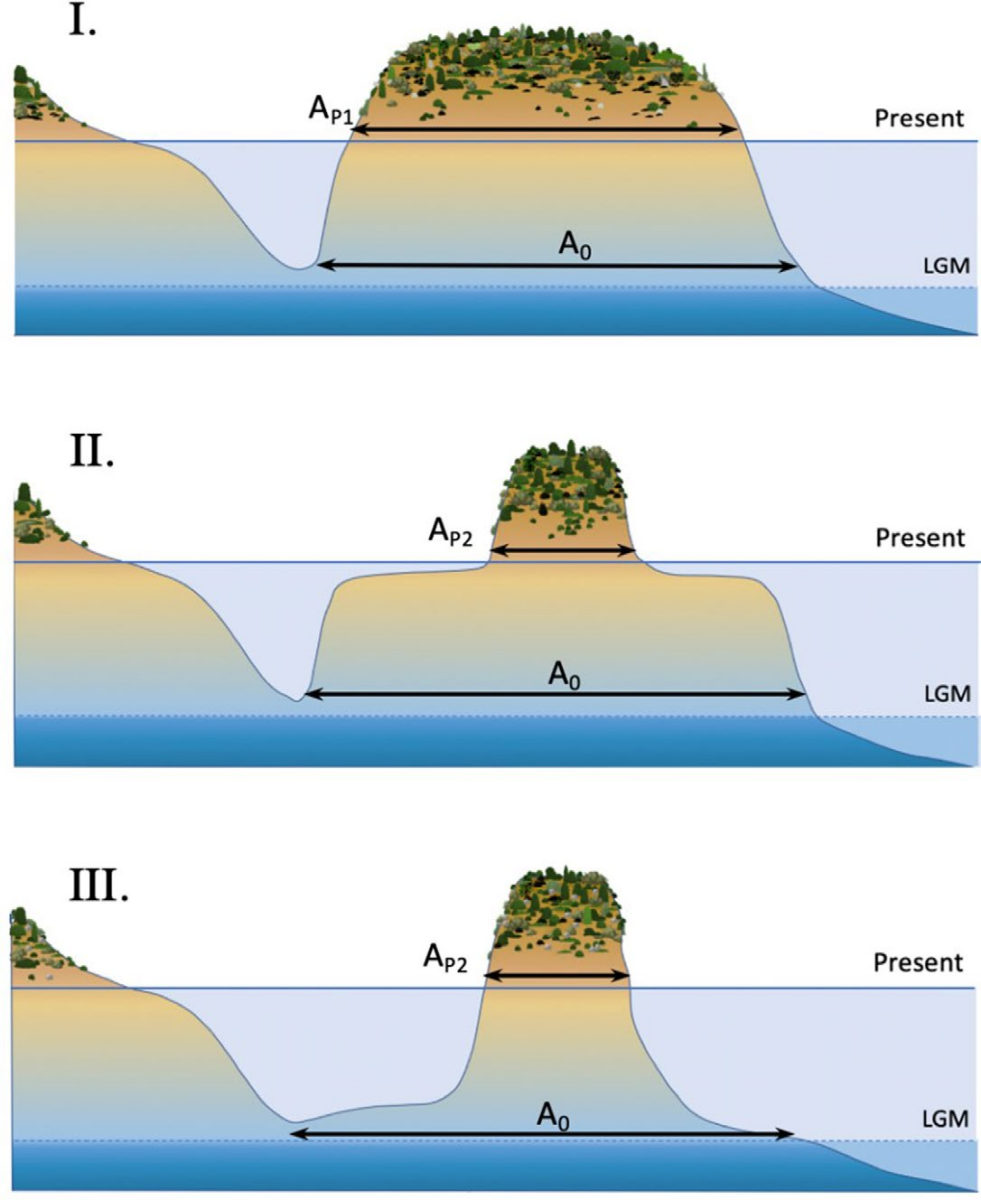

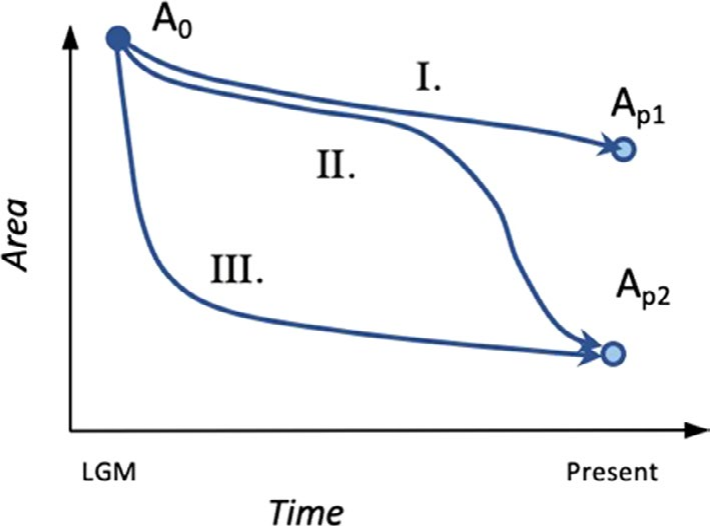

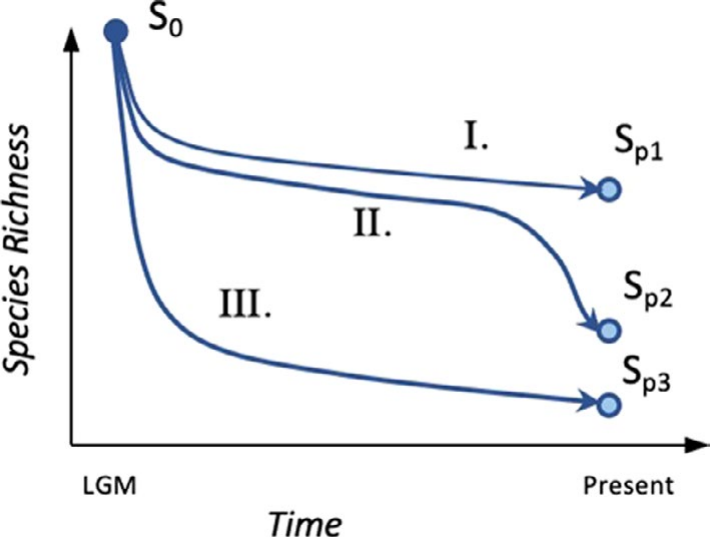

In the Mediterranean Sea, the Aegean archipelago is located between the Greek and the Anatolian peninsulas and is one of the largest archipelagos on Earth (Blondel et al., 2010). Its complex geological history and high environmental heterogeneity contribute to its high biodiversity and endemism, thus rendering it an ideal stage for biogeographic studies (Sfenthourakis & Triantis, 2017; Strid, 2016). Unsurprisingly, its biogeography has been studied intensively (e.g., Hausdorf & Hennig, 2005; Itescu et al., 2020; Panitsa et al., 2010; Triantis et al., 2018). Although these studies provide crucial biogeographic insights on diversity patterns in continental island settings, it has remained an open question how species diversity and chorology are influenced by continental islands that were connected during the glacial sea‐level low stands (land‐bridge islands) and continental islands that always had remained islands (“true islands” sensu Simaiakis et al., 2017). The combined effect of area change, fragmentation, and connectivity driven by late Quaternary sea‐level changes on insular species diversity has never been investigated for the Aegean archipelago, although such a combined analysis is crucial to interpret the relevant biogeographic processes that drive species diversity and evolutionary patterns (see Kougioumoutzis & Tiniakou, 2014; Norder et al., 2019). Moreover, the influence of past connections to the mainland on island species–area relationships (ISARs) has never been assessed (Fattorini, 2017; Triantis et al., 2008, 2012). Since we have recently quantified the paleogeographic change of islands in the Aegean Sea (Simaiakis et al., 2017) and its biota are well studied, this setting represents an ideal study system to investigate the influence of late Quaternary sea‐level fluctuations on native and endemic species richness.

**FIGURE 1:**
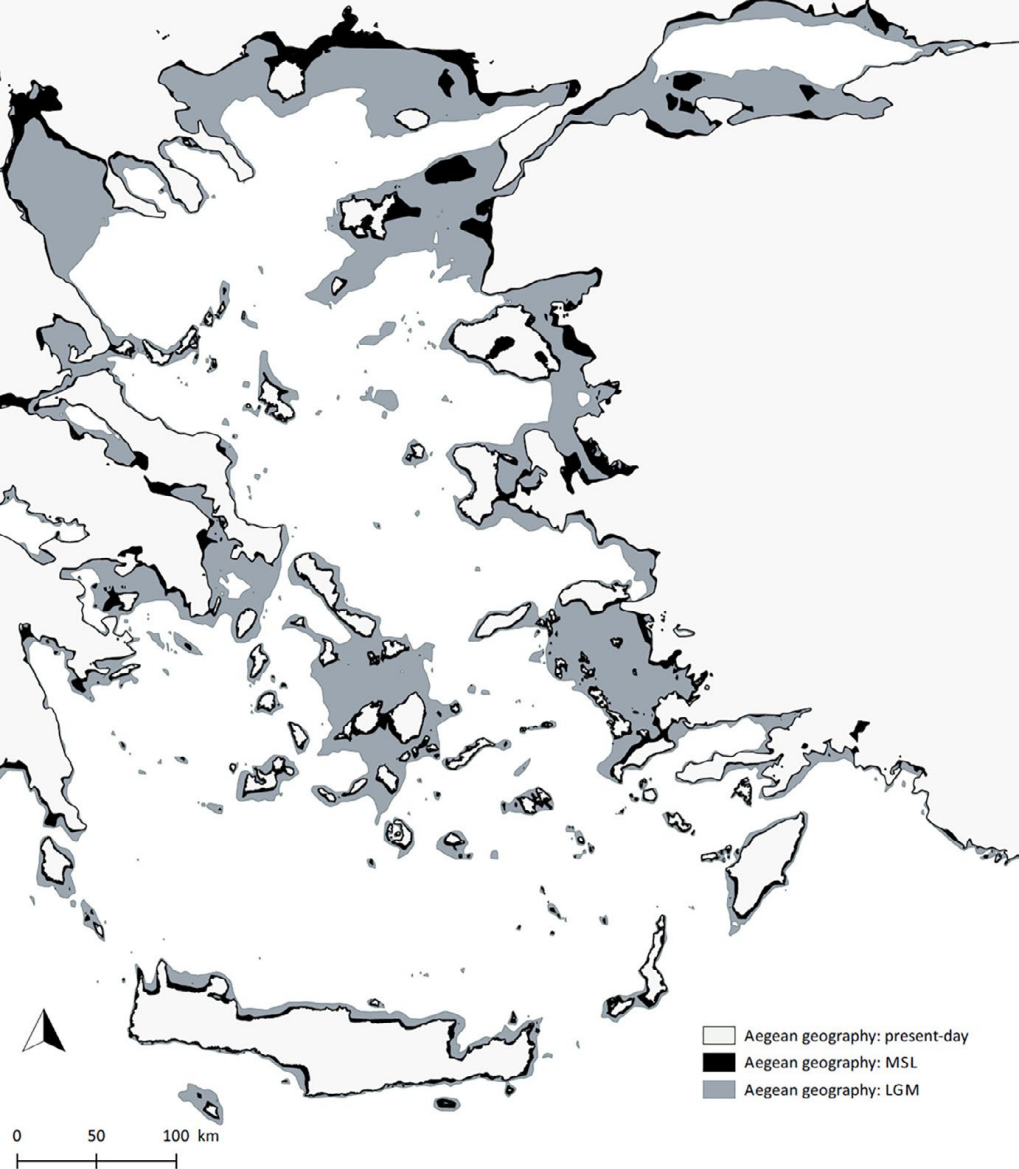
Map of the current geography of the Aegean archipelago with estimated land surfaces at median sea level (MSL, black) and during the Late Glacial Maximum (LGM, gray)

Our aim is to investigate the combined impact of current, as well as past, island area and connectivity on the insular species diversity of four well‐studied taxonomic groups (angiosperms, reptiles, butterflies, and centipedes) in the Aegean archipelago. Our first hypothesis (H_1_) is that more native species occur per area unit on land‐bridge islands than on true islands (sensu Simaiakis et al., 2017), reflecting the higher establishing rates of native species on land‐bridge islands (Simaiakis et al., 2017). Our second hypothesis (H_2_) is that endemism is negatively influenced by past connections to the mainland, as allopatric speciation is suppressed by repetitive genetic exchanges with continental populations. Our third hypothesis (H_3_) concerns the effect of the duration of the archipelagic configurations as a result of sea‐level drop on richness patterns. On oceanic islands, the median archipelago configuration largely explains richness patterns of single‐island endemics, rather than the extreme and short‐lasting Last Glacial Maximum (LGM) configuration (Norder et al., 2019). We hypothesize that on land‐bridge islands too, the median geographic configuration (representative of the last glacial–interglacial cycles) has largely influenced patterns of species richness. In contrast, the extreme LGM configuration should not have provided the time needed for speciation to occur and is therefore not expected to have significantly influenced patterns of species richness. To test our two first hypotheses, we analyze the combined effects of island type (land‐bridge vs. true islands) and change in area and isolation on species richness of different taxa and chorotypes. We investigate H_3_ by analyzing species richness patterns in relation to three paleogeographic settings: (a) current; (b) during LGM; and (c) the median sea level (MSL) during the last glacial–interglacial cycle (Figure 1).

## 2 METHOD

### 2.1 The Aegean archipelago

The Aegean lies at the convergence of three tectonic plates (Anatolian, African, and Eurasian) and has therefore developed major fault systems, resulting in the formation of the South Aegean Volcanic Arc (Higgins, 2009). However, even though plate tectonics have strongly shaped the paleo‐evolution of the Aegean archipelago, its recent history has been mainly affected by Pleistocene climatic fluctuations (Sakellariou & Galanidou, 2016). Compared to the magnitude of the geographic changes caused by the sea‐level oscillations during the last ca. 120 Ka BP (comprising the last glacial–interglacial cycle; Georgopoulou et al., 2015), the importance of the tectonic changes occurring at the same time scale is mostly negligible given our aim and scope (Simaiakis et al., 2017). During the LGM (26.5–19 Ka BP), the global sea level was ~135 m lower than present and locally >140 m lower in the Mediterranean (Clark et al., 2009; Lambeck & Purcell, 2005; Lambeck et al., 2014). Subsequent sea‐level rise caused dramatic changes in the Aegean basin with most of the marine transgression observed during 16–11 Ka BP, when sea‐level rise rates were 12 m/1 Ka (Simaiakis et al., 2017). This led to a major reduction of total island area by ca. 70% in the Aegean basin and a rapid increase in the number of islands by fragmentation. Large islands (>20 km^2^) were rapidly shrinking and becoming isolated during that time (Simaiakis et al., 2017). Islands emerged near the coast of Turkey and Greece that were formerly peninsulas and the Cycladic paleo‐island fragmented into the Cyclades islands group (Figure 1).

### 2.2 Species richness data

We compiled datasets of species richness for angiosperms, butterflies, centipedes, and reptiles from published work (Table A1.1). Data matrices of angiosperms and reptiles were used to investigate patterns of both native and endemic species richness as their high rate of endemism allowed for such statistical analysis. Due to the paucity of data related to centipede and butterfly endemic species and the low number of islands hosting them, we focused on native species for our model selection analyses for these two taxa, and only considered endemics in our exploratory analyses.

**FIGURE 2:**
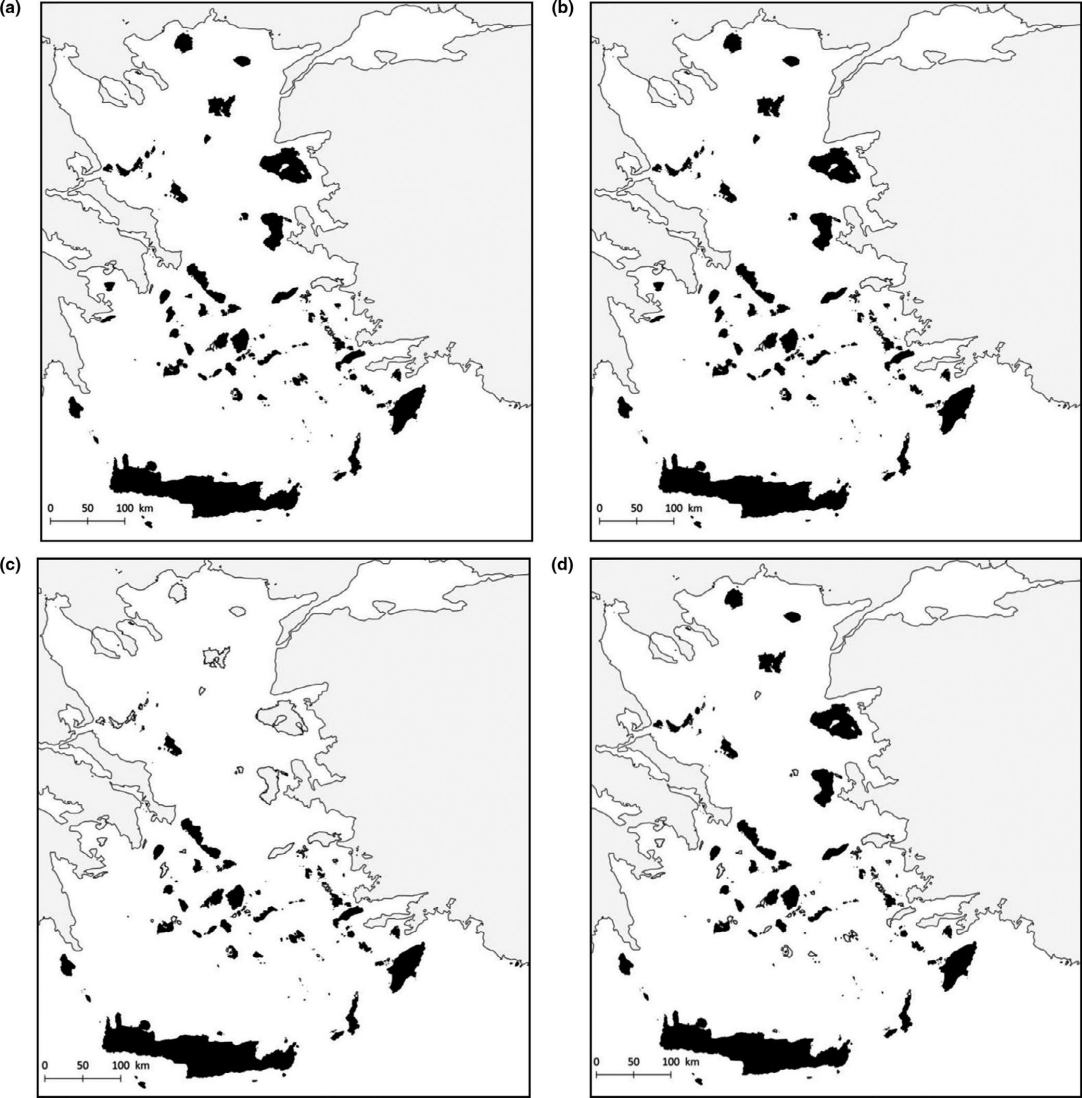
Maps of the islands used for the analysis of the four taxa included in this study. A, angiosperms; B, reptiles; C, centipedes; D, butterflies

**FIGURE 3:**
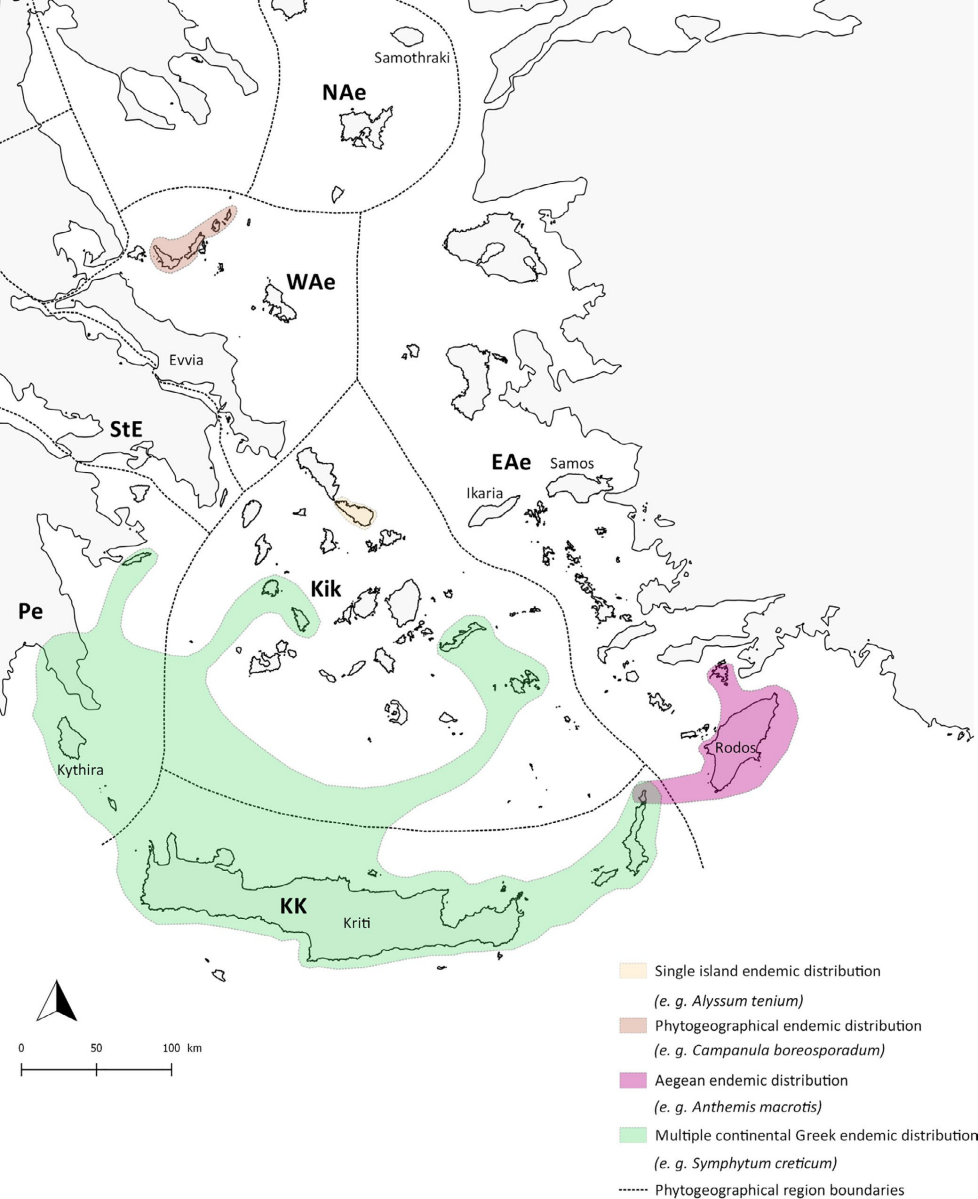
Map of the islands cited in the text, the phyto‐geographic regions of the Aegean, and the distribution of the four angiosperm species in the Aegean archipelago, illustrating the characteristics of their respective chorotypes. NAe, North Aegean; WAe, West Aegean; EAe, East Aegean; StE, Sterea Ellas; Kik, Cyclades; KK, Crete

We compiled a plant matrix for 70 Aegean islands (Figure 2A), including a total of 3,246 native angiosperm taxa (species and subspecies). The angiosperms are a well‐studied group in the Aegean, and we compiled data on five chorotypes (native nonendemics, multiple continental Greek endemics, Aegean endemics, phyto‐region endemics, and single‐island endemics) and two combined chorotypes (all endemics and multiple island endemics) based on an extensive bibliographical database for the Aegean archipelago (see Appendix S1 in Kougioumoutzis et al., 2017). Examples of the distribution of the 4 endemics chorotypes are presented in Figure 3. The “native nonendemics,” sometimes termed “natives,” are a widely spread taxonomic group occurring both on the Aegean islands and on the mainland across the Mediterranean (2,673 taxa), ~40% of which reached the Aegean islands as a result of human action in prehistoric times (Greuter, 1971, 1979; Kougioumoutzis et al., 2020, modern introductions were excluded). The “multiple continental Greek endemics” (GE) are species exclusively occurring both on the Greek mainland and the Aegean islands (689 taxa). The “Aegean endemics” (AE) are found on multiple islands but not on the mainland (91 taxa). The “phyto‐region endemics” (PE) are restricted to one or multiple islands located within the same Aegean phyto‐geographic region sensu Strid and Tan (1997—384 taxa). Finally, the single‐island endemics (SIEs) are endemics occurring exclusively on one Aegean island (292 taxa). We also combined chorotypes to compare general trends of endemics with nonendemics. We combined both phyto‐region and Aegean endemics to form “multiple islands endemics” (MIEs) sensu *stricto* (450 taxa) and combined all‐endemic chorotypes into an “all‐endemics group” (E_ALL_).

We compiled a database for the distributions of reptiles on 70 islands of the Aegean archipelago (Figure 2, B). Data were gleaned from the extensive literature (Valakos et al., 2008, Poulakakis et al., 2014, Bellati et al., 2015, Marzahn et al., 2016, Kornilios et al., 2020, Spilani et al., 2019, Thanou et al., 2020). Only peer‐reviewed published records, or records backed up by specimens deposited in scientific collections, were used. When considering native taxa, we excluded island populations that had been likely introduced by humans in the recent past. This includes island occurrences where published information reports on very recent arrival (e.g., *Stellagama* on Karpathos, Grano & Cattaneo, 2019) or where molecular phylogeographic work has demonstrated recent, likely human‐assisted arrival in the region (e.g., *Hemidactylus turcicus*). As for angiosperms, we recognized endemic status both at the species and subspecies level. This was based on taxonomic information published in peer‐reviewed journals, accepted over the years by the herpetological community, and ideally also supported by molecular analyses. Number of native species varies greatly between 1 (Gavdopoula) and 22 (Kos) and number of endemic species varied between 0 (numerous islands) and 5 (e.g., Milos).

A similar matrix was compiled for 56 Aegean islands (Figure 2, C) and 70 centipede species (see Simaiakis et al., 2004, 2005). Two chorotypes were distinguished: “native” and “endemics” (65 and 5 species, respectively). The number of native species varies between 2 (Dragonada) and 34 (Crete), whereas most islands (82%) host no endemic species.

Finally, a total of 117 species of butterflies from 37 Aegean islands (Figure 2D) were compiled into a matrix, based on the data published by Pamperis (2019). Distribution maps of species were georeferenced using ArcMap v.10.6, and the presence of species on the islands was recorded. Six species occur only on one Aegean island (SIE). The rest 111 species are distributed on other Aegean islands and/or (at least) in mainland Greece and Turkey.

### 2.3 Paleo‐island geography

We used the work of Simaiakis et al. (2017) to estimate insular areas and distance to the mainland at MSL and LGM sea level. In this earlier work, the Aegean archipelago was reconstructed based on a geophysical model of relative sea‐level change that uses generalized sea‐level equations accounting for hydro‐isostatic adjustments and applied on a topographic and bathymetric grid with a resolution of 30 arc‐seconds (Simaiakis et al., 2017). For the construction of the MSL scenario, we aimed to get close to a global eustatic median sea‐level stand of 65 m below present for the last nine glacial–interglacial cycles, following the method from Norder et al. (2019), building on the works of Tzedakis et al. (2012) and Bintanja et al. (2005). We assumed that the regional geophysical effects affecting this eustatic MSL stand were minimal and therefore used the geographic setting at 11 ka BP (latest occurrence of 65 m below present sea level) as estimate for MSL scenario. For the LGM setting, we based our analysis on the reconstruction at 21 Ka BP. Islands smaller than the surface of a grid cell (1 km^2^) were excluded from our analysis, and islands separated by a distance smaller than the grid size (1 km^2^) were aggregated in the paleogeographic reconstruction to obtain a conservative estimate of the fragmentation dynamics occurring in the system (*cf*. Rijsdijk et al., 2014). All geographic data were processed in ArcGIS 10.2.2. After aggregation, island areas and Euclidean shore‐to‐shore distance to the mainland (without correction for satellite islets) for the current, MSL (11 Ka BP), and LGM (21 Ka BP) configurations were used to calculate the difference between current and past areas as well as current and past distances (Table A 1.1), with the aim to use these variables in further inferential analysis (*cf*. method of Weigelt et al., 2016). In addition, to investigate the biogeographic effects of past fragmentation from the mainland, we classified islands into two different groups (Table A 1.2): those that remained isolated from the mainland, referred to as “true islands” (sensu Simaiakis et al., 2017) versus those with an episodic connection to the mainland during the LGM (“LGM land‐bridge islands”) or under MSL scenario (“MSL land‐bridge islands”). For land‐bridge islands (distance = 0 at median sea level or Late Glacial Maximum), the area considered is that of the island at the time step (1 Kya) preceding the connection to the mainland.

### 2.4 Exploratory and inferential analysis

ISARs were fitted using the logarithmic transformation of the Arrhenius power model (Carey et al., 2020). We compared the ISARs of different taxa/chorotypes using adjusted‐R^2^ values as a measure of their goodness of fit. As the models have the same number of fitted parameters, the R^2^ are directly comparable, without modification (Triantis et al., 2005). We also calculated the mean area‐adjusted species richness (number of species divided by insular area) of true islands and land‐bridge islands for the native and all‐endemic chorotypes of all taxa. We assessed whether the difference of mean area‐adjusted species richness observed between the two type of islands was significant using ANOVA.

To investigate the impact of past geographic changes on current diversity, we compared the performance of three alternative GLMMs in explaining species richness. The models were built identically for all taxa and chorotypes and corresponded to the geographic setting of the Aegean under three scenarios: present‐day, LGM, and MSL. The present‐day model (null model) consisted of present‐day area, Euclidean shore‐to‐shore distance to the mainland, spatial autocorrelation as fixed effect, and phyto‐geographic region as random effect to account for biogeographic affinity. The models for the paleogeographic settings (LGM or MSL) included the same predictors as the null model plus the following metrics representing the changes in insular geography compared to the present‐day setting: area loss, increase of Euclidean distance to the mainland and island type (“true” vs. *“*land‐bridge”). We fitted these three sets of predictors using the species richness of all aforementioned taxa and chorotypes as response variables. All models were fitted using a log‐link function and Poisson distribution for the error term (lmerTest package, Kuznetsova et al., 2017). All variables with *p*‐values > 0.1 were excluded from the models to obtain a set of “suggestive, but inconclusive” predictors (Murtaugh, 2014) before refitting with the remaining variables. Multicollinearity was addressed by computing the variance inflation factors (VIF) of the predictors and removing variables with VIF > 2.5 (Dormann et al., 2013). Finally, we identified the models that provided most explanatory potential using multiple measures of goodness of fit: the corrected Akaike information criterion (AICc), Bayesian information criterion (BIC), and leave‐one‐out cross‐validated (loocv) pseudo‐R^2^. In addition, in accordance with Nakagawa and Schielzeth (2013), we calculated the difference between conditional (R^2^c, fit with random effect) and marginal (R^2^m, fit without random effect) R^2^ to assess how much additional variance biogeographic affinity (random factor) explained in our models (MuMIn package, Barton, 2017). All analyses were performed in R version 3.4.2 (R core team, 2018). Crete was diagnosed as an outlier in several GLMMs using residual plots of residuals versus leverage (data not shown), but was still included in the analysis due to its biogeographic importance in the Aegean.

## 3 RESULTS

### 3.1 Geographic changes in the Aegean

The geographic reconstructions based on Simaiakis et al. (2017) confirm that the geography of the Aegean archipelago changed dramatically throughout the last climatic cycle of the Quaternary (Figure 1). The current configuration and the LGM setting represent two extremes of the sea‐level fluctuations (highest now and lowest during the LGM). The changes in areas calculated reflect this pattern: Insular area loss between the LGM and the present is systematically greater compared to the difference between MSL and the present (Table A 1.1). Similarly, the increase of distance between islands and mainland is greater for the LGM–present comparison compared to MSL–present (Table A 1.1). Finally, fewer islands were connected to the mainland (land‐bridge islands) under MSL configuration compared to the LGM (Table A 1.2).

### 3.2 Exploratory and inferential analyses

**FIGURE 4:**
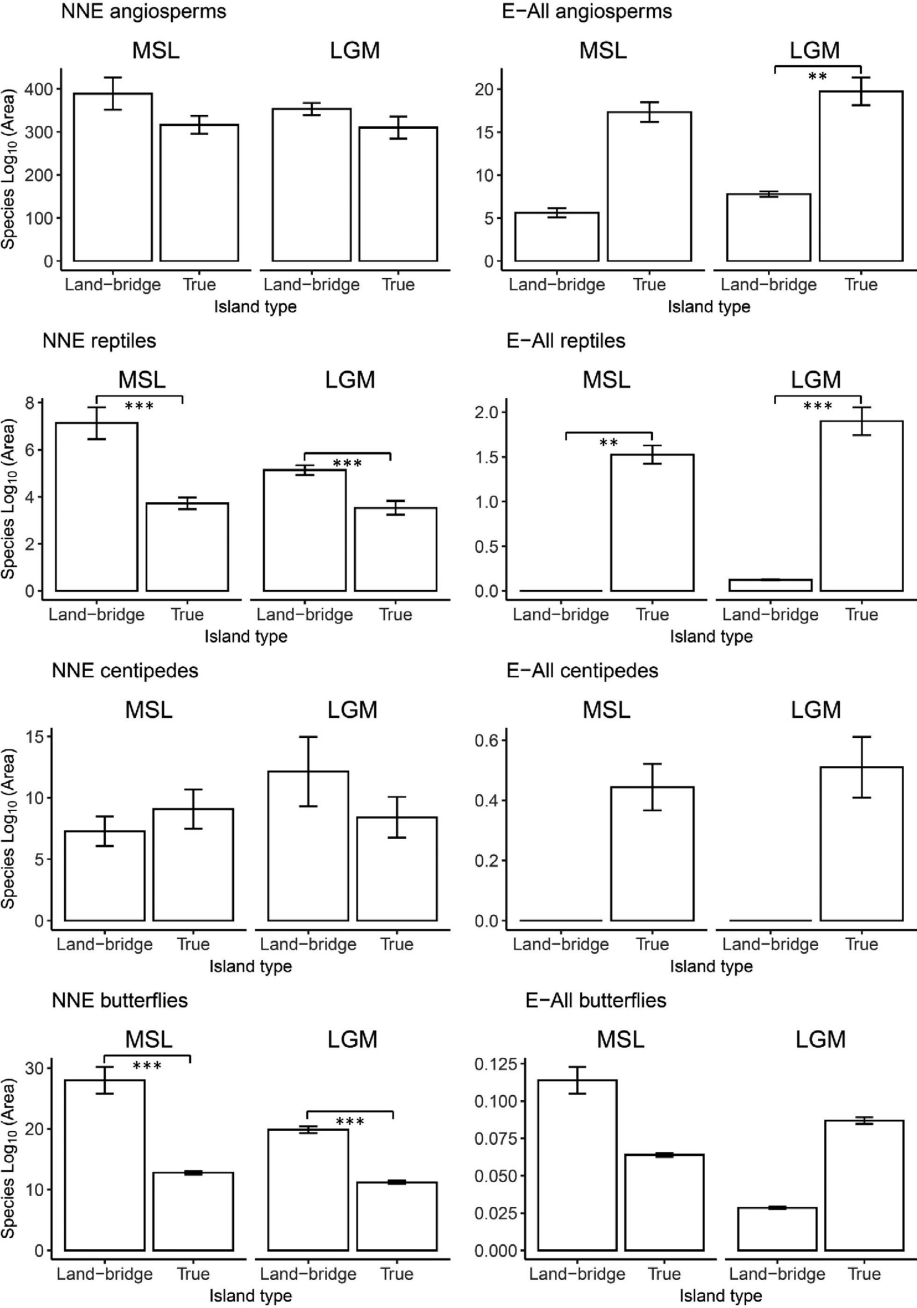
Bar plot of the averaged, area‐adjusted insular species richness (species richness / log10 of insular area) for the two island types studied for the Last Glacial Maximum (LGM) and Median Sea Level (MSL). Error bars report standard error on the mean. NNE = Native nonendemics; E‐All = All endemics. Significance was addressed using ANOVA tests. **: *p*‐value < .01, ***: *p*‐value < .001

The proportion of variance explained by ISARs varied greatly between taxa, chorotypes, and island types; adjusted‐R^2^ ranged −0.05 for endemic reptiles on land‐bridge islands from to 0.79 for native nonendemic angiosperms (Table 1). ISARs for endemic subsets were generally supported by lower adjusted‐R^2^ compared to native nonendemic subsets. Similarly, adjusted‐R^2^ was lower when fitting the ISARs on subsets of land‐bridge islands compared to true islands. ISARs slopes (*z*‐values) also varied greatly between taxa, chorotypes, and island types, and ranged from −0.01 for native nonendemic reptiles to 0.37 for angiosperm SIEs on true islands (Table 1, Figure A2). Land‐bridge islands appear to host more native taxa than true islands, whereas the opposite trend was observed regarding endemic taxa which were more abundant on true islands than on land‐bridge islands, as indicated by the comparison of the area‐adjusted means of species richness (Figure 4) and the ISARs (intercept higher on land‐bridge islands for native nonendemics and lower for endemics—Figure A 2).

**TABLE 1:** Slope and adjusted‐R2 of the log_10_‐transformed island species area relationship curves

Taxon	Chorotype	z‐values			Adjusted R^2^		
		Land‐bridge islands	True Islands	Both	Land‐bridge islands	True Islands	Both
Angiosperms	NNE	0.30***	0.33***	0.33***	0.73	0.78	0.79
	All‐E	0.35*	0.32***	0.26***	0.26	0.51	0.25
	PE	0.25*	0.21***	0.14*	0.16	0.18	0.05
	AE	0.13	0.25***	0.14*	−0.01	0.33	0.06
	GE	0.38*	0.33***	0.30***	0.22	0.52	0.33
	SIEs	0.30*	0.37***	0.34***	0.20	0.45	0.39
	MIEs	0.19	0.25***	0.14*	0.03	0.29	0.05
Reptiles	NNE	0.30***	0.29***	0.33***	0.57	0.51	0.56
	All‐E	0.02	0.10**	−0.01	−0.05	0.14	−0.01
Centipedes	NNE	−0.03	0.18***	0.15***	−0.11	0.37	0.26
Butterflies	NNE	0.29*	0.31***	0.35***	0.42	0.52	0.48

Note

NNE is native nonendemic species, All‐E is all endemics, PE is phyto‐region endemics, AE is Aegean endemics, GE is Greek island endemics, SIEs is single‐island endemics, MIEs is Multiple island endemic.

Asterisks indicate the significance level: * *p*‐value ≤ .05; ** *p*‐value ≤ .01; and *** *p*‐value ≤ .001.

**FIGURE 5:**
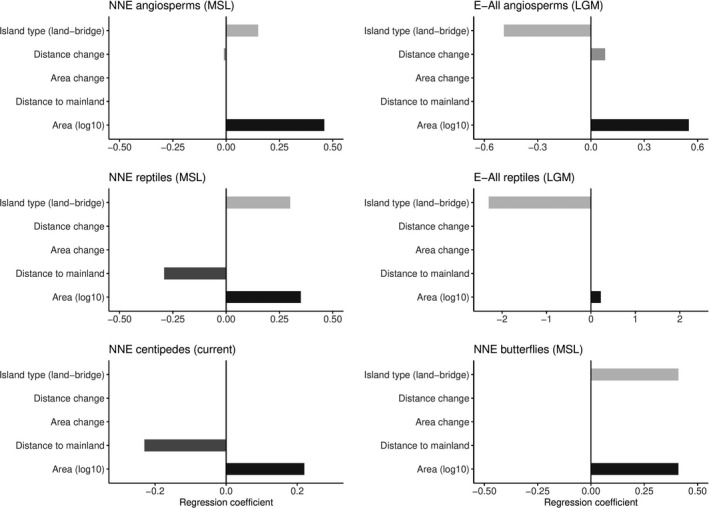
Value and sign of the regression coefficients of the predictors of the best models selected (see Table 1) for the different taxa and some of the chorotypes analyzed. The colors are used to assist in finding corresponding variables in the different subplots. Note the different ranges on the x‐axis for the different subplots. NNE, Native nonendemics; E‐All, All endemics

The proportion of variance explained (here estimated using the loocv‐R^2^) by the GLMMs ranged from 14% (angiosperm SIEs) to 84% (butterflies’ native nonendemics—Table 2) and was overall greatest for butterflies, followed by angiosperms, reptiles, and finally centipedes. Biogeographic affinity (accounted for by the random effect and inferred from the difference between R^2^c and R^2^m) explained a large proportion of variance for butterflies and for all angiosperm endemic categories (10%–78%—Table 2), but not for the native angiosperms, centipedes, and reptiles (Table 2). Current area was the most important predictor in almost all models (Table 2; Figure 5). Among the variables accounting for paleogeographic changes, only island type was consistently selected across taxa and chorotypes, and its effect size was comparable to current area. Area loss and increase of distance to the mainland were only rarely selected as significant predictors of species richness, and when they were, their magnitude of effect was minor compared to current area. In addition to reflecting the current area, native angiosperm species richness was also influenced by MSL island type, and to a minor extent by the increase of distance to the mainland compared to MSL. LGM‐related variables (mostly island type) emerged as important predictors for all‐endemic angiosperm chorotypes, except for the Greek endemics and the SIEs, for which no effect of paleogeography was detected (Table 2; Figure 5). The effect of island type was opposite on native and endemic chorotypes whereby land‐bridge islands hosted more native species than true islands, but much less endemics. GLMMs for reptile chorotypes yielded similar results; native species richness was influenced by MSL island type in addition to the current geography, whereas the LGM model was clearly superior for endemic species richness, also revealing a strong effect of island type (Table 2). For native nonendemic centipedes, no influence of paleogeography was detected, whereas the native nonendemic butterfly species richness was best explained by a combination of the current area and MSL island type, with an over‐representation in land‐bridge islands (Table 2).

**TABLE 2:** Selected Generalized Linear Mixed Model (GLMM) models for each chorotype with overall best performance in bold

Taxon	Chorotype	Sea‐level	Model	Model (predictors: p‐value ≤ 0.1 | VIF ≤ 2.5)	AICc	BIC	loocv‐R^2^	R^2^c ‐ R^2^m
			(m.)					
Angiosperms (*n* = 70)	Native nonendemic	Present	1	logApr ‐ Dpr + S‐A + prov	2,377	2,387	0.75	0,03
		**MSL**	**2**	**0.46 logApr ‐ 0.01 dDmed + 0.15 typemed (P) + S‐A + prov**	**2,298**	**2,310**	0.75	0,03
		LGM	3	logApr ‐ Dpr + typelgm (P) ‐ 0.01 S‐A + prov	2,332	2,344	0.75	0,02
	All endemics	Present	4	logApr + Dpr +S‐A + prov	647	657	0.38	0,45
		MSL	5	logApr + Dpr +dDmed + typemed +S‐A + prov	651	664	0.39	0,43
		**LGM**	**6**	**0.55 logApr + 0.08 dDlgm ‐ 0.49 typelgm (P) + S‐A + prov**	**629**	**641**	**0.43**	0,36
	Phyto‐region endemics	Present	7	logApr + Dpr +S‐A + prov	351	361	0.48	0,84
		MSL	8	logApr + typemed +S‐A + prov	344	355	0.47	0,83
		**LGM**	**9**	**0.39 logApr ‐ 0.58 typelgm (P) ‐ S‐A + prov**	**340**	**351**	**0.50**	0,78
	Aegean endemics	Present	10	logApr + S‐A + prov	490	498	0.42	0,71
		MSL	11	logApr + dDmed +typemed + S‐A + prov	465	477	0.43	0,68
		**LGM**	**12**	**0.50 logApr + 0.13 Dpr ‐ 0.14 dAlgm ‐ 0.57 typelgm (P) + S‐A + prov**	**464**	478	**0.46**	0,55
	Multiple continental Greek endemics	**Present**	**13**	**0.63 logApr ‐ S‐A + prov**	**368**	**376**	**0.60**	0,44
		MSL	14	logApr + S‐A + prov	368	376	0.60	0,44
		LGM	15	logApr + S‐A + prov	368	376	0.60	0,44
	Single‐island endemics	**Present**	**16**	**1.51 logApr + 0.35 Dpr + S‐A + prov**	**228**	**239**	**0.14**	0,10
		MSL	17	logApr + Dpr +S‐A + prov	228	239	0.14	0,10
		LGM	18	logApr + S‐A + prov	228	239	0.14	0,10
	Multiple island endemics	Present	19	logApr + Dpr +S‐A + prov	571	581	0.45	0,79
		MSL	20	logApr + dDmed ‐ typemed (P) + S‐A + prov	552	564	0.45	0,77
		**LGM**	**21**	**0.49 logApr + 0.13 Dpr ‐ 0.10 dAlgm ‐ 0.58 typelgm (P) + S‐A + prov**	**537**	**551**	**0.48**	0,68
Reptiles (*n* = 70)	Native nonendemics	Present	22	logApr ‐ Dpr + S‐A + prov	323	333	0.57	0,00
		**MSL**	**23**	**0.35 logApr ‐ 0.29 Dpr + 0.30 typemed (P) + S‐A + prov**	**321**	333	**0.61**	0,00
		LGM	24	logApr ‐ Dpr + S‐A + prov	323	333	0.57	0,00
	All endemics	Present	25	logApr + S‐A + prov	217	225	0.55	0,26
		MSL	26	logApr + S‐A + prov	217	225	0.55	0,26
		**LGM**	**27**	**0.22 logApr ‐ 2.3 typelgm (P) + S‐A + prov**	**197**	**207**	**0.60**	0,00
Centipedes (*n* = 56)	Native nonendemics	**Present**	**28**	**0.22 logApr ‐ 0.23 Dpr + S‐A + prov**	**297**	**306**	**0.37**	0,01
		MSL	29	logApr ‐ Dpr + S‐A + prov	297	306	0.37	0,01
		LGM	30	logApr ‐ Dpr + S‐A + prov	297	306	0.37	0,01
Butterflies (*n* = 37)	Native nonendemics	Present	31	logApr ‐ Dpr + S‐A + prov	287	293	0.62	0,32
		**MSL**	**32**	**0.41 logApr + 0.41 typemed (P) + S‐A + prov**	**270**	**277**	**0.84**	0,35
		LGM	33	logApr ‐Dpr + typelgm (P) + S‐A + prov	280	287	0.68	0,17

Note

Model performance was assessed based on corrected Akaike information criterion (AICc), Bayesian information criterion (BIC), and leave‐one‐out cross‐validated (loocv) pseudo‐R^2^. The difference between conditional and marginal R^2^ (R^2^c and R^2^m, respectively) was used to assess the impact of the random effect of the models (phyto‐geographic region to which the island belongs). In each of these models, variables with p‐values higher than 0.1 or variance inflation factor (VIF) higher than 2.5 were removed. Meaning of acronyms: MSL = Median Sea Level; LGM = Last Glacial Maximum; *S‐A* = spatial autocorrelation; *prov* = phyto‐geographic region; *logApr* = logarithm of current area; *Dpr* = current shortest distance to the mainland; *dDmed* = increase in distance to the mainland compared to median sea level; *dAmed* = insular area loss compared to median sea level; *typemed* = island type (land‐bridge island or true island) based on median sea level; *dDlgm* = increase in distance to the mainland compared to LGM sea level; *dAlgm* = insular area loss compared to LGM sea level; *typelgm* = island type based on LGM sea level. Sample size information is provided in the first column (Taxon), and it remains the same for all submodels within this group.

## 4 DISCUSSION

Late Quaternary sea‐level changes have left their mark on island species richness patterns (e.g. Norder et al., 2019; Weigelt et al., 2016), following theoretical expectations (Fernández‐Palacios et al., 2015). Recently, the question arose whether LGM paleogeographic configurations or those more representative of the Pleistocene better explain endemic species richness (Norder et al., 2019). On oceanic islands, the intermediate sea‐level configuration seems to have shaped SIE diversity patterns (Norder et al., 2019). Our study aimed to investigate the possibility that different patterns might be observed on continental‐shelf island systems. Here, we start by discussing whether and how past connections to the mainland—one of the most distinctive features of continental islands systems compared to oceanic ones—have had an impact on the diversity of native nonendemic or endemic species richness. Then, we discuss current, MSL, and LGM influences on the patterns of species richness observed and compared our results with those obtained in oceanic settings. Finally, we complement our discussion by taxa‐specific considerations on the importance of biogeographic affinities.

### 4.1 Native nonendemic species richness

Our first hypothesis predicted that native nonendemic species should be more abundant on land‐bridge islands than on true islands because of their increased connectivity with the mainland throughout the last glacial–interglacial cycle resulting in higher establishment rate of continental species. Our results corroborate this hypothesis in the cases of angiosperms, reptiles, and butterflies, whereas the richness of native nonendemic centipedes was not influenced by past connections to the mainland (Table 2). For native angiosperms, GLMMs had high predictive capacity (Table 2) which is in line with Kreft et al. (2008), Kagiampaki et al. (2011), Kougioumoutzis and Tiniakou (2014), or Valli et al. (2019) (*i.e*., 82%–95%). Results for this taxon group indicated that native nonendemic species richness was significantly higher on MSL land‐bridge islands compared to true islands, which is also apparent in the slightly higher position of the ISAR curve of the land‐bridge islands compared to true islands (Figure A2). In the “best” GLMM, the regression coefficient of the current area was three times higher than that of island type, suggesting only a modest influence of past connections to the mainland on the current richness of native nonendemic angiosperm species. Native nonendemic butterfly species richness, though, was strongly influenced by past mainland connections as indicated by a regression coefficient for island type as high as that of current area. As for angiosperms, MSL rather than LGM island type was selected in our analysis. However, we would like to point out that the butterfly dataset only included 3 MSL land‐bridge islands, versus 12 for the LGM period, so that in this specific case, the selection of the “best” time frame might be less robust than suggested by the AICc and BIC values (Table 2). Either way, our results indicate that the species richness of native butterflies is strongly influenced by past connections to the mainland, which directly contradicts the results of Dennis et al. (2000), according to which butterfly diversity did not reflect historical influences in the Aegean. Similarly, for reptiles, the effect of island type indicated an over‐representation of native species on land‐bridge islands and was of the same order of magnitude as the current area. That effect was apparent in the higher intercept of the ISAR of land‐bridge islands compared to true islands. This may be explained in part by the fact that because reptiles are relatively poor over‐water dispersers, once isolated, species communities will undergo community relaxation progressively losing species over time (Diamond, 1972; Foufopoulos & Ives, 1999; Newmark, 1987). Finally, native centipede species richness did not reflect any influence of paleogeography. Furthermore, the proportion of variance explained by the current model was relatively low (37%, Table 2), which may indicate that other factors (e.g., habitat heterogeneity) are more influential than insular area or isolation for this taxon (Triantis et al., 2005, Simaiakis and Martinez‐Morales, 2005). In summary, results show that MSL land‐bridge islands host proportionally more native species than true islands for all taxa except centipedes.

### 4.2 Endemic species richness

Our second hypothesis predicted that land‐bridge islands should host proportionally fewer endemic species than true islands because episodic connections to the mainland would have created windows of gene flow between previously isolated insular populations and their mainland relatives, thereby counteracting their genetic differentiation in allopatry. The results obtained for the GLMM selection of both taxa formally analyzed with regard to endemics diversity (angiosperms and reptiles) strongly corroborate this hypothesis, though for angiosperms the effect was chorotype dependent.

Except for the SIEs and the multiple continental Greek endemics, the patterns of species richness of all‐endemic chorotypes showed strong imprints of the LGM geographic configuration through the connections established to the mainland during that period of low sea level. Indeed, island type was the variable accounting for paleogeography most consistently selected in our analysis. Furthermore, its regression coefficient was higher than that of the current area, indicating that the effect of LGM geography was at least as influential as the current setting in shaping patterns of endemic angiosperm species richness. This result is confirmed by the lower intercept of endemic angiosperm chorotypes (except aforementioned) on land‐bridge islands compared to true islands. A highly similar pattern was observed for reptiles: Land‐bridge islands hosted significantly fewer endemics species than true islands, as reflected in the output of both model selection and ISARs intercepts. Although the centipedes and butterflies’ datasets were not formally analyzed with regard to patterns of endemic species richness, a rough examination of the distribution of the few endemic species present in the study system fits with the aforementioned patterns of depletion on land‐bridge islands. Indeed, none of the 9 land‐bridge islands included in the centipedes dataset hosted any endemic species. Similarly, only 1 out of the 12 land‐bridge islands of butterflies dataset hosted 1 endemic species.

### 4.3 Current, median, and LGM influences

Our third working hypothesis predicted that MSL rather than LGM insular configurations should have influenced patterns of species richness as the former is more representative of the geographic setting over the last glacial–interglacial cycle. Overall, our results validate this hypothesis for the native chorotypes of angiosperms, reptiles, and butterflies, but not for the endemic chorotypes of these taxa nor for centipedes. It is noteworthy to point that GLMM selection most clearly indicates that current island geography is highly influential in determining the species richness of all taxa and chorotypes except endemic reptiles. More specifically, current area was consistently included in all models with comparatively high regression coefficients, whereas current distance to the mainland was only included in the models of a few taxa and chorotypes (Aegean and multiple island angiosperm endemics, native reptiles and native centipedes—Table 2). By comparison to the effect of current area, the respective influence of MSL‐ and LGM‐related variables was more taxon and chorotype dependent.

The maximum, yet short‐lasting connectivity achieved during the LGM (Figure 1; Sakellariou & Galanidou, 2016) has negatively affected angiosperm endemic diversity (except SIEs and mainland Greek endemics), suggesting that the LGM spatial configuration of the Aegean archipelago has left its imprint on species’ distribution, richness, and evolutionary patterns (e.g., Kougioumoutzis et al., 2017; Poulakakis et al., 2015). The reptile endemic species richness follows a highly similar pattern, thus also pointing toward an influence of short‐lasting episodes of increased connectivity with the mainland as counteracting speciation process. Finally, reptile, butterfly, and angiosperm native nonendemic species richness were all affected by MSL island type. Altogether, this suggests that unlike endemic species richness, native diversity is more affected by the longer lasting geographic configuration, being partly in line with the flickering connectivity hypothesis (Flantua & Hooghiemstra, 2018). The fact that angiosperm SIEs are largely unaffected by past configurations differs from observations made in oceanic insular settings, where MSL configurations were highly influential (Norder et al., 2019). We believe that, in a system of islands as highly interconnected as the Aegean, topographic complexity and environmental heterogeneity might be the main driver of SIEs diversification (e.g., Kallimanis et al., 2011; Kougioumoutzis et al., 2020, 2021; Lazarina et al., 2019). Similarly, multiple continental Greek endemics were unaffected by the variables accounting for paleogeographic changes included in the analysis, thus indicating that other processes might shape the patterns of diversity of this chorotype. As these species could have originated from the Greek mainland, we suggest that a negative longitudinal trend (reflecting the pattern of migration from the continent) could govern their distribution. Altogether, the fact that paleogeography had a heterogeneous effect on different chorotypes of the taxon highlights the importance of distinguishing between these chorotypes when addressing biogeographic questions.

It is noteworthy to mention that we observed that some results regarding the “best” time frame selected for island type were highly influenced by the inclusion of Crete in the analysis, as it acted as a high‐leverage outlier. For example, removing Crete from the GLMMs selection procedure for the native angiosperm resulted in selecting the LGM rather than MSL island type.

### 4.4 Biogeographic affinity

Biogeographic affinity (i.e., the random effect in our models) explained only a small fraction of the variance for the native nonendemic angiosperms, nonendemic and endemic reptiles, and nonendemic centipedes. The high proportions of generalist species and of species with high dispersal abilities in native nonendemic chorotypes might be responsible for this pattern. However, we did not investigate the issue further as it goes beyond the scope of this study. For angiosperms, the lack of influence of biogeographic affinity can partially be attributed to the fact that a large portion (~40%) of the present Aegean flora has reached the Aegean islands due to human action in prehistoric or early historic times (Greuter, 1971, 1979; Kougioumoutzis et al., 2020). For reptiles, the lack of effect of biogeographic affinity could result from similar levels of diversity in the various source communities “seeding” the different islands east and west of the Aegean Trench (Foufopoulos et al., 2011).

A large share of variance of the models of all angiosperm endemic chorotypes as well as native nonendemic butterflies was explained by biogeographic affinity. For endemic angiosperms, this is in line with previous studies stating that in the Aegean, bioregionalization is primarily a result of the region's complex paleogeographic history (Iliadou et al., 2020; Kougioumoutzis et al., 2017). This is also due the high proportion of narrowly ranged species and consequently of high species turnover in Aegean island plant communities (e.g., Iliadou et al., 2020): Nearly ~45% of the endemic taxa occurring in the Aegean are SIEs, the vast majority of which occur in Crete and Evvia (Panitsa et al., 2018). Other factors such as climate and geodiversity probably play an important role in shaping current endemic diversity patterns throughout the Aegean, as was observed for the central (Kougioumoutzis & Tiniakou, 2014), eastern (Panitsa et al., 2010; Panitsa & Tzanoudakis, 2010) and southern (Kagiampaki et al., 2011) Aegean islands. In addition, favorable climatic conditions most probably permitted a relict flora to persist in the southern (i.e., Crete, Karpathos, and Rodos) and partly eastern (e.g., Ikaria: Christodoulakis, 1996, 1996) Aegean archipelago (Runemark, 1969, 1971). In topographically complex islands, some of the old MIEs formed neo‐endemic SIEs through allopatric speciation (Runemark, 1969, 1971, Bittkau & Comes, 2005, 2009, Comes et al., 2008, Jaros et al., 2018; see also figure 7 in Kougioumoutzis et al., 2021 regarding neo‐endemism centers in the Aegean). Geographic isolation through sea‐level oscillations may have supported the recent diversification of neo‐endemic species, especially in the central Aegean where several nonadaptive radiations occurred (e.g., *Campanula*, *Nigella*, *Erysimum*—e.g., Comes et al., 2008, Jaros et al., 2018). Specifically, episodes of high insular fragmentation occurred repetitively over the last 2 My (every glacial‐interglacial interval for ~20 ka), disrupting the longer lasting glacially connected state and leading to cumulative genetic divergence between populations according to the flickering connectivity hypothesis (Aguilée et al., 2009; Flantua & Hooghiemstra, 2018). One prominent example for this is the differentiation of the *Nigella arvensis* species complex due to nonadaptive radiation and random genetic drift resulting from several vicariant events during the Pliocene/Pleistocene (Bittkau & Comes, 2005, 2009; Jaros et al., 2018).

According to Dennis et al. (2000), the percentage of endemic butterflies is very low in the Aegean in compliance with our findings. Also, common species live in the center of the Aegean (i.e., the Cyclades) whereas peripheral islands (close to Greek and Turkish mainland) host both rare and common species (Dennis et al., 2000), which might explain the high influence of biogeographic affinities for this taxon. Additionally, butterflies may largely follow the phyto‐region compartmentalization of the Aegean since they are habitat and resource specialists, with many groups being food–plant specialists (Dennis et al., 2000; Ricklefs & Lovette, 1999).

### 4.5 Continental‐shelf and oceanic archipelagos

The geographic setting of the LGM is an extreme configuration that was represented only sporadically and for short time‐periods throughout the Pleistocene, with only 2% of the last 800 Ka BP estimated to have a similarly low sea level (Norder et al., 2019). Therefore, the duration of these episodes may have been too short to have had a significant impact on the diversity and composition of insular biota on oceanic islands (Heaney et al., 2013; Norder et al., 2019; Porter, 1989). However, our results show that in a continental‐shelf archipelago, patterns of endemic species richness of multiple taxa (angiosperms and reptiles) were strongly and negatively influenced by connections with the mainland that occurred during the LGM. We explain this as the result of extreme connectivity that was achieved during the LGM between the expanded Aegean true islands, land‐bridge islands, and the surrounding continental land mass. This connectivity with the mainland, even though short‐lasting, could have promoted the over‐representation of native butterfly (and angiosperm) species by enabling high rates of establishment on land‐bridge islands for species from the mainland during episodic connections. The same increased connectivity would on the other hand have been detrimental to ongoing speciation processes in land‐bridge islands, by enabling new gene flow from populations of mainland relatives, thus resulting in the strong patterns of endemic under‐representation observed for angiosperms and reptiles. Alternatively, the invasion of island communities by mainland taxa during LGM‐like conditions could have led to the extinction of competitively inferior island endemics (Capula et al., 2002).

The ΙSAR z‐values for total angiosperm richness fall into the z‐value range for continental island settings. On the other hand, the endemic richness z‐values are much lower than those on oceanic islands, pointing to reduced endemism on continental islands. We also observed that the high degree of Aegean island fragmentation by Pleistocene sea‐level oscillations led to MIEs sharing much more islands (>5) than is observed for oceanic archipelagos. The multiple island angiosperm endemism manifested in the Aegean today is indeed largely shaped by the fragmentation of large landmasses into smaller islands by the present high sea level. It remains to be tested in how far maximum connectivity conditions are exclusively related to the lowest short‐lasting sea‐level stand, or whether for most biota maximum connectivity was reached earlier and lasted longer than the LGM. Clearly, this depends on the geometry and depth of the basin under study, influencing the sea‐level thresholds at which island area and connectedness change significantly (Norder et al., 2019). We can conclude there are two crucial differences between oceanic and continental islands: (a) in a continental setting, episodic connections with the mainland have profound effects on the biota of land‐bridge islands and (b) the rate and magnitude of area loss, as well as the degree of fragmentation, is far greater in continental compared to oceanic systems. As such, metrics of area loss or distance increase such as the ones used in Weigelt et al. (2016) or Norder et al. (2019) are of little relevance when disentangling biogeographic patterns in continental island systems. Indeed, most islands included in our dataset were several times larger during the LGM than now, because they belonged to larger paleo‐islands at that moment. Such large losses of area cannot be expected to be reflected in patterns of species richness the same way area contraction would be on oceanic islands and is indeed not reflected in the outputs of GLMM selection, which showed area loss and distance increase to have a minor and occasional influence on diversity patterns only.

## 5 CONCLUSIONS

Our study highlighted the importance of past sea‐level changes in shaping species richness patterns in the Aegean, with MSL or LGM influences being relevant depending on the taxon and chorotype. On the one hand, LGM connections to the mainland were linked to strong endemic under‐representation in land‐bridge islands, a pattern which differs from previous findings according to which MSL shaped patterns of endemic species richness in oceanic systems. On the other hand, compared to true islands, MSL land‐bridge islands hosted significantly more native species of all taxa except centipedes, potentially reflecting an increased rate of establishment over a long period of time on these islands. Furthermore, area loss and increase of distance to the mainland had low overall performance in model selection procedure, probably as a result of the magnitude of geographic changes endured by islands following the fragmentation of large paleo‐landmasses. Continental systems thus hold ample evidence of the effect of paleogeography on the species richness, speciation, and biogeographic patterns of the Aegean islands. The noted principal differences in geographic and evolutionary mechanisms between oceanic and continental islands make statistical assessments of endemic species data of combined oceanic and continental islands fundamentally problematic, and studies involving both systems should explicitly incorporate their differences in statistical models.

## CONFLICT OF INTEREST

The authors have no conflict of interests.

## AUTHOR CONTRIBUTION

**Cyril Hammoud:** Conceptualization (equal); Data curation (equal); Formal analysis (lead); Investigation (equal); Methodology (equal); Project administration (equal); Visualization (lead); Writing‐original draft (lead); Writing‐review & editing (equal). **Konstantinos Kougioumoutzis:** Conceptualization (equal); Data curation (equal); Investigation (equal); Methodology (equal); Resources (lead); Writing‐original draft (equal); Writing‐review & editing (lead). **Kenneth F. Rijsdijk:** Conceptualization (equal); Data curation (equal); Formal analysis (supporting); Investigation (equal); Methodology (equal); Resources (equal); Writing‐original draft (supporting); Writing‐review & editing (equal). **Stylianos M. Simaiakis:** Conceptualization (equal); Investigation (equal); Methodology (supporting); Resources (lead); Writing‐original draft (supporting); Writing‐review & editing (equal). **Sietze J. Norder:** Conceptualization (supporting); Investigation (supporting); Methodology (supporting); Writing‐review & editing (equal). **Johannes Foufopoulos:** Resources (equal); Validation (supporting); Writing‐review & editing (supporting). **Elisavet Georgopoulou:** Resources (equal); Validation (supporting); Writing‐review & editing (supporting). **Emiel E. Van Loon:** Conceptualization (equal); Formal analysis (equal); Investigation (supporting); Methodology (equal); Visualization (supporting); Writing‐original draft (supporting); Writing‐review & editing (equal).

## AUTHOR'S CONTRIBUTIONS

CH, KFR, SJN, and EEVL: Workflow design. CH: manuscript writing. KK: Plant data compilation. SMS: Centipede data compilation. JF: Reptile data compilation. EG: Butterfly data compilation. All authors contributed equally to manuscript writing and interpretation of results.

## Supporting information

Supplementary Material

Supplementary Material

Supplementary Material

Supplementary Material

Supplementary Material

Supplementary Material

Supplementary Material

Supplementary Material

Supplementary Material

Supplementary Material

Supplementary Material

## Data Availability

GIS shapefiles of insular geography at the different time frames, as well as species richness matrixes: Dryad doi.org/10.5061/dryad.dfn2z350z.
