# Supplementary material for: Past connections with the mainland structure patterns of insular species richness in a continental‐shelf archipelago (Aegean Sea, Greece)
Source: Ecol Evol. 2021 Mar 29;11(10):5441–58. doi: 10.1002/ece3.7438 (PMC8131802; doi:10.1002/ece3.7438)
Supplement: Supplementary file 1 — Supplementary Material [file ECE3-11-5441-s008.docx]

**Supplementary Materials.**

Appendix 1:

Table A1.1

Current and past insular area and distance to the mainland, as well as species richness for the different taxa and chorotypes analysed. MSL = Median Sea Level; LGM = Last Glacial Maximum; NAe = North Aegean; WAe = West Aegean; EAe = East Aegean; Kik = Cyclades; KK = Crete. NNE is native non-endemic species, All-E is all endemics, PE is phyto-region endemics, AE is Aegean endemics, GE is Greek island endemics, SIEs is single island endemics, MIEs is Multiple island endemic.

| Island | Current area (km^2^) | Area at MSL (km^2^) | Area at LGM (km^2^) | Current distance (km) | MSL distance (km) | LGM distance (km) | Phyto-geographic region | Angiosperm | | | | | | | Reptiles | | Centipedes | | Butterflies | |
| --- | --- | --- | --- | --- | --- | --- | --- | --- | --- | --- | --- | --- | --- | --- | --- | --- | --- | --- | --- | --- |
|  |  |  |  |  |  |  |  | NNE | E-All | PE | AE | GE | SIEs | MIEs | NNE | E-All | NNE | E-All | NNE | E-All |
| *Agathonisi* | 13.44 | 36.39 | 54.13 | 17.89 | 13.00 | 0.00 | EAe | 364 | 6 | 0 | 6 | 0 | 0 | 6 | 4 | 0 | NA | NA | NA | NA |
| *Agios Efstratios* | 42.02 | 74.41 | 233.17 | 82.98 | 79.20 | 0.00 | NAe | 441 | 2 | 0 | 1 | 1 | 0 | 1 | 4 | 0 | NA | NA | NA | NA |
| *Aigina* | 84.22 | 210.65 | 359.54 | 6.71 | 4.12 | 0.00 | Pe | 813 | 28 | 0 | 2 | 26 | 0 | 2 | 7 | 0 | NA | NA | NA | NA |
| *Alatonisi* | 1.83 | 1.83 | 8.97 | 53.45 | 21.21 | 2.24 | EAe | NA | NA | NA | NA | NA | NA | NA | NA | NA | 3 | 0 | NA | NA |
| *Alimia* | 7.47 | 12.47 | 105.01 | 37.44 | 38.01 | 35.85 | EAe | 136 | 7 | 2 | 4 | 1 | 0 | 6 | 6 | 2 | NA | NA | NA | NA |
| *Alonnisos* | 113.08 | 123.85 | 372.58 | 40.82 | 27.00 | 0.00 | WAe | 546 | 18 | 6 | 3 | 9 | 0 | 9 | 9 | 2 | NA | NA | 40 | 0 |
| *Amorgos* | 121.33 | 179.61 | 295.05 | 105.38 | 76.49 | 49.82 | Kik | 731 | 59 | 12 | 29 | 17 | 1 | 41 | 5 | 3 | 8 | 0 | 20 | 0 |
| *Anafi* | 38.52 | 80.45 | 258.77 | 140.13 | 101.64 | 93.48 | Kik | 516 | 36 | 3 | 23 | 9 | 1 | 26 | 2 | 2 | 5 | 0 | NA | NA |
| *Andros* | 441.78 | 734.17 | 7616.97 | 12.08 | 10.77 | 5.39 | Kik | 949 | 46 | 8 | 13 | 22 | 3 | 21 | 12 | 4 | 12 | 0 | NA | NA |
| *Antikythira* | 19.68 | 34.00 | 161.09 | 59.54 | 58.25 | 38.21 | Pe | 312 | 24 | 2 | 8 | 13 | 1 | 10 | 3 | 2 | 10 | 0 | 29 | 0 |
| *Antimilos* | 8.81 | 15.98 | 23.48 | 92.85 | 92.10 | 77.00 | Kik | 130 | 5 | 1 | 2 | 2 | 0 | 3 | 3 | 3 | NA | NA | 9 | 0 |
| *Antiparos* | 61.69 | 966.62 | 7616.97 | 110.54 | 105.95 | 5.39 | Kik | 398 | 14 | 3 | 7 | 4 | 0 | 10 | 7 | 3 | 6 | 0 | NA | NA |
| *Astypalaia* | 95.87 | 182.34 | 360.01 | 80.23 | 40.80 | 33.62 | Kik | 532 | 36 | 3 | 21 | 11 | 1 | 24 | 3 | 2 | 8 | 0 | NA | NA |
| *Chalki* | 27.20 | 43.15 | 105.01 | 45.12 | 45.12 | 35.85 | EAe | 421 | 18 | 7 | 9 | 2 | 0 | 16 | 5 | 2 | 11 | 0 | 18 | 0 |
| *Chios* | 843.92 | 952.13 | 952.13 | 7.07 | 0.00 | 0.00 | EAe | 1183 | 30 | 4 | 12 | 12 | 2 | 16 | 20 | 0 | NA | NA | 64 | 1 |
| *Dia* | 11.88 | 20.04 | 39.61 | 210.95 | 203.67 | 200.11 | KK | 97 | 23 | 10 | 8 | 4 | 1 | 18 | 2 | 1 | 3 | 2 | NA | NA |
| *Donoussa* | 13.66 | 29.05 | 7616.97 | 118.34 | 89.83 | 5.39 | Kik | 340 | 25 | 7 | 12 | 6 | 0 | 19 | 3 | 2 | 5 | 0 | NA | NA |
| *Dragonada* | 2.87 | 16.35 | 31.17 | 181.96 | 159.31 | 158.82 | KK | 259 | 46 | 18 | 24 | 4 | 0 | 42 | 2 | 1 | 2 | 1 | NA | NA |
| *Elasa* | 4.04 | 6.35 | 10906.46 | 180.85 | 163.17 | 87.24 | KK | NA | NA | NA | NA | NA | NA | NA | NA | NA | 11 | 1 | NA | NA |
| *Fokionisi Megalo* | 1.59 | 182.34 | 360.01 | 89.89 | 40.80 | 33.62 | Kik | NA | NA | NA | NA | NA | NA | NA | NA | NA | 5 | 0 | NA | NA |
| *Folegandros* | 32.35 | 54.43 | 7616.97 | 131.47 | 127.42 | 5.39 | Kik | 435 | 31 | 9 | 14 | 7 | 1 | 23 | 4 | 3 | 11 | 0 | 11 | 0 |
| *Gavdopoula* | 1.77 | 7.58 | 358.21 | 180.94 | 180.55 | 169.16 | KK | 199 | 13 | 5 | 3 | 4 | 1 | 8 | 1 | 1 | 3 | 2 | NA | NA |
| *Gavdos* | 32.72 | 67.60 | 358.21 | 191.09 | 187.54 | 169.16 | KK | 457 | 19 | 8 | 7 | 3 | 1 | 15 | 3 | 2 | 4 | 2 | 6 | 0 |
| *Gioura* | 11.05 | 21.64 | 178.07 | 59.48 | 57.58 | 3.61 | WAe | 374 | 23 | 6 | 5 | 12 | 0 | 11 | 2 | 2 | NA | NA | NA | NA |
| *Gyali* | 8.42 | 12.69 | 27.02 | 19.10 | 8.25 | 6.32 | EAe | NA | NA | NA | NA | NA | NA | NA | NA | NA | 16 | 0 | NA | NA |
| *Gyaros* | 17.55 | 34.24 | 7616.97 | 38.95 | 38.01 | 5.39 | Kik | 222 | 8 | 1 | 1 | 6 | 0 | 2 | 3 | 2 | NA | NA | NA | NA |
| *Ikaria* | 254.68 | 327.96 | 504.25 | 50.70 | 18.25 | 0.00 | EAe | 919 | 38 | 5 | 15 | 8 | 10 | 20 | 9 | 1 | NA | NA | 40 | 0 |
| *Ios* | 108.45 | 160.75 | 7616.97 | 145.01 | 134.00 | 5.39 | Kik | 580 | 23 | 3 | 11 | 9 | 0 | 14 | 6 | 5 | 8 | 0 | 27 | 0 |
| *Irakleia* | 18.12 | 33.21 | 7616.97 | 146.01 | 127.19 | 5.39 | Kik | 347 | 23 | 6 | 11 | 6 | 0 | 17 | 5 | 4 | NA | NA | NA | NA |
| *Kalymnos* | 248.26 | 272.57 | 293.03 | 16.49 | 4.00 | 0.00 | EAe | 753 | 19 | 4 | 10 | 5 | 0 | 14 | 14 | 0 | 14 | 0 | 36 | 0 |
| *Kandelioussa* | 3.28 | 3.28 | 7.27 | 38.59 | 17.03 | 16.03 | EAe | NA | NA | NA | NA | NA | NA | NA | NA | NA | 5 | 0 | NA | NA |
| *Karpathos* | 393.83 | 471.05 | 831.28 | 85.15 | 85.15 | 84.53 | KK | 938 | 94 | 30 | 46 | 12 | 6 | 76 | 5 | 3 | 13 | 0 | 28 | 1 |
| *Kasos* | 66.71 | 149.74 | 831.28 | 140.94 | 135.00 | 84.53 | KK | 532 | 48 | 16 | 25 | 7 | 0 | 41 | 4 | 3 | 12 | 0 | 10 | 0 |
| *Kastellorizo* | 15.13 | 15.13 | 16.56 | 3.00 | 2.83 | 0.00 | EAe | NA | NA | NA | NA | NA | NA | NA | NA | NA | 19 | 0 | NA | NA |
| *Kea* | 130.38 | 166.65 | 239.21 | 19.65 | 13.60 | 0.00 | Kik | 632 | 22 | 4 | 8 | 10 | 0 | 12 | 7 | 0 | 7 | 0 | 25 | 0 |
| *Keros* | 15.09 | 30.34 | 7616.97 | 138.45 | 109.66 | 5.39 | Kik | 273 | 25 | 7 | 14 | 4 | 0 | 21 | 3 | 2 | NA | NA | NA | NA |
| *Kimolos* | 48.66 | 344.91 | 710.79 | 101.07 | 97.58 | 73.98 | Kik | 492 | 26 | 5 | 11 | 10 | 0 | 16 | 9 | 5 | NA | NA | NA | NA |
| *Kinaros* | 4.56 | 9.21 | 63.78 | 82.39 | 59.51 | 23.85 | Kik | 147 | 11 | 1 | 8 | 1 | 1 | 9 | 2 | 2 | NA | NA | NA | NA |
| *Kithnos* | 99.43 | 138.82 | 229.74 | 37.64 | 32.53 | 5.39 | Kik | 573 | 32 | 5 | 14 | 13 | 0 | 19 | 8 | 2 | NA | NA | NA | NA |
| *Kos* | 288.10 | 438.91 | 438.91 | 7.00 | 0.00 | 0.00 | EAe | 1012 | 12 | 2 | 7 | 2 | 1 | 9 | 22 | 0 | 14 | 0 | NA | NA |
| *Koufonisi* | 4.18 | 21.04 | 10906.46 | 221.47 | 203.08 | 87.24 | KK | 248 | 13 | 6 | 5 | 2 | 0 | 11 | 3 | 3 | 4 | 1 | NA | NA |
| *Kounoupoi* | 3.98 | 182.34 | 360.01 | 80.52 | 40.80 | 33.62 | Kik | NA | NA | NA | NA | NA | NA | NA | NA | NA | 9 | 0 | NA | NA |
| *Koutsomytis* | 1.51 | 182.34 | 360.01 | 82.76 | 40.80 | 33.62 | Kik | NA | NA | NA | NA | NA | NA | NA | NA | NA | 11 | 0 | NA | NA |
| *Kriti* | 8264.6 | 9208.39 | 10906.46 | 95.08 | 93.98 | 87.24 | KK | 1466 | 325 | 44 | 59 | 50 | 172 | 103 | 7 | 4 | 34 | 5 | 42 | 7 |
| *Kyra Panagia* | 24.76 | 38.34 | 178.07 | 62.37 | 47.13 | 3.61 | WAe | 411 | 18 | 6 | 6 | 6 | 0 | 12 | 3 | 2 | NA | NA | 7 | 0 |
| *Kythira* | 277.23 | 365.72 | 646.13 | 13.45 | 7.07 | 6.00 | Pe | 763 | 56 | 4 | 8 | 41 | 3 | 12 | 12 | 1 | 7 | 0 | 41 | 0 |
| *Leipsoi* | 15.87 | 46.91 | 118.79 | 36.67 | 32.39 | 0.00 | EAe | 506 | 12 | 0 | 8 | 4 | 0 | 8 | 7 | 0 | 8 | 0 | NA | NA |
| *Lesvos* | 1636.7 | 2007.52 | 2007.52 | 9.00 | 7.81 | 0.00 | EAe | 1438 | 13 | 3 | 2 | 6 | 2 | 5 | 20 | 0 | NA | NA | 83 | 0 |
| *Levitha* | 9.12 | 19.98 | 63.78 | 65.28 | 46.40 | 23.85 | Kik | 170 | 14 | 0 | 10 | 4 | 0 | 10 | 2 | 2 | NA | NA | NA | NA |
| *Limnos* | 477.03 | 879.62 | 1794.26 | 58.24 | 26.25 | 0.00 | NAe | 674 | 10 | 0 | 1 | 8 | 1 | 1 | 11 | 0 | NA | NA | 42 | 0 |
| *Milos* | 196.57 | 344.91 | 710.79 | 103.37 | 97.58 | 73.98 | Kik | 736 | 35 | 4 | 16 | 15 | 0 | 20 | 10 | 5 | 9 | 0 | 24 | 0 |
| *Mykonos* | 85.80 | 144.22 | 7616.97 | 86.05 | 84.63 | 5.39 | Kik | 591 | 21 | 4 | 6 | 11 | 0 | 10 | 9 | 5 | 7 | 0 | 19 | 0 |
| *Naxos* | 430.17 | 966.62 | 7616.97 | 120.25 | 105.95 | 5.39 | Kik | 1055 | 57 | 9 | 23 | 20 | 5 | 32 | 10 | 4 | 9 | 0 | 33 | 0 |
| *Nisyros* | 41.28 | 55.84 | 76.27 | 16.64 | 15.23 | 13.34 | EAe | 522 | 8 | 1 | 3 | 3 | 1 | 4 | 9 | 0 | 17 | 0 | 19 | 0 |
| *Ofidousa* | 3.79 | 5.78 | 360.01 | 109.12 | 69.64 | 33.62 | Kik | NA | NA | NA | NA | NA | NA | NA | NA | NA | 6 | 0 | NA | NA |
| *Paros* | 238.19 | 966.62 | 7616.97 | 107.62 | 105.95 | 5.39 | Kik | 772 | 37 | 10 | 14 | 13 | 0 | 24 | 10 | 4 | 8 | 0 | 28 | 0 |
| *Patmos* | 34.22 | 67.88 | 104.40 | 48.80 | 33.00 | 0.00 | EAe | 404 | 6 | 1 | 4 | 0 | 1 | 5 | 7 | 0 | 8 | 0 | 21 | 0 |
| *Paximada* | 1.53 | 16.35 | 31.17 | 179.49 | 159.31 | 158.82 | KK | NA | NA | NA | NA | NA | NA | NA | NA | NA | 3 | 1 | NA | NA |
| *Polyaigos* | 18.12 | 344.91 | 710.79 | 109.18 | 97.58 | 73.98 | Kik | 246 | 11 | 2 | 2 | 6 | 1 | 4 | 7 | 5 | NA | NA | NA | NA |
| *Pontikousa* | 2.43 | 4.03 | 360.01 | 102.42 | 62.36 | 33.62 | Kik | NA | NA | NA | NA | NA | NA | NA | NA | NA | 8 | 0 | NA | NA |
| *Psara* | 40.04 | 75.04 | 173.51 | 61.27 | 19.00 | 12.00 | EAe | 356 | 6 | 0 | 4 | 2 | 0 | 4 | 4 | 0 | NA | NA | NA | NA |
| *Pserimos* | 14.63 | 438.91 | 438.91 | 8.06 | 0.00 | 0.00 | EAe | 273 | 0 | 0 | 0 | 0 | 0 | 0 | 8 | 0 | 12 | 0 | NA | NA |
| *Rineia* | 14.06 | 50.46 | 7616.97 | 83.44 | 82.07 | 5.39 | Kik | 399 | 12 | 3 | 4 | 5 | 0 | 7 | 4 | 3 | NA | NA | NA | NA |
| *Ro* | 2.60 | 4.27 | 22.01 | 5.00 | 5.00 | 0.00 | EAe | NA | NA | NA | NA | NA | NA | NA | NA | NA | 14 | 0 | NA | NA |
| *Rodos* | 1407.6 | 1615.73 | 1982.09 | 18.03 | 17.09 | 13.93 | EAe | 1272 | 53 | 10 | 24 | 11 | 8 | 34 | 16 | 4 | 21 | 1 | 54 | 0 |
| *Samothraki* | 180.51 | 225.11 | 250.23 | 35.36 | 29.07 | 0.00 | NAe | 1262 | 33 | 0 | 3 | 17 | 13 | 3 | 17 | 0 | NA | NA | 57 | 0 |
| *Schoinousa* | 8.13 | 56.13 | 7616.97 | 146.82 | 116.40 | 5.39 | Kik | 372 | 28 | 7 | 11 | 10 | 0 | 18 | 4 | 3 | 5 | 0 | NA | NA |
| *Serifos* | 74.09 | 102.86 | 176.48 | 62.80 | 58.52 | 34.01 | Kik | 588 | 24 | 7 | 7 | 10 | 0 | 14 | 9 | 3 | 9 | 0 | 11 | 0 |
| *Sifnos* | 77.38 | 110.49 | 161.28 | 86.83 | 82.42 | 57.71 | Kik | 660 | 39 | 8 | 14 | 16 | 1 | 22 | 7 | 4 | 9 | 0 | 22 | 0 |
| *Sikinos* | 41.74 | 72.12 | 7616.97 | 142.27 | 137.54 | 5.39 | Kik | 460 | 37 | 8 | 19 | 9 | 1 | 27 | 4 | 3 | 8 | 0 | NA | NA |
| *Skantzoura* | 6.23 | 15.31 | 31.55 | 59.55 | 42.05 | 8.06 | WAe | 261 | 6 | 0 | 2 | 4 | 0 | 2 | 2 | 2 | NA | NA | NA | NA |
| *Skiathos* | 47.33 | 72.32 | 72.32 | 4.12 | 0.00 | 0.00 | WAe | 674 | 12 | 1 | 0 | 10 | 1 | 1 | 12 | 0 | NA | NA | 49 | 0 |
| *Skopelos* | 125.96 | 141.33 | 372.58 | 22.00 | 6.08 | 0.00 | WAe | 645 | 17 | 4 | 3 | 9 | 1 | 7 | 8 | 2 | NA | NA | 23 | 0 |
| *Skyropoula* | 6.05 | 6.29 | 38.89 | 26.17 | 26.17 | 26.17 | WAe | NA | NA | NA | NA | NA | NA | NA | NA | NA | 2 | 0 | NA | NA |
| *Skyros* | 206.93 | 317.85 | 586.30 | 33.62 | 34.13 | 30.41 | WAe | 811 | 34 | 4 | 9 | 19 | 2 | 13 | 9 | 3 | 14 | 0 | 49 | 0 |
| *Stroggyli* | 3.50 | 3.50 | 4.80 | 1.41 | 1.41 | 0.00 | EAe | NA | NA | NA | NA | NA | NA | NA | NA | NA | 13 | 0 | NA | NA |
| *Symi* | 58.00 | 102.60 | 125.78 | 8.00 | 8.00 | 5.00 | EAe | 565 | 17 | 4 | 10 | 1 | 2 | 14 | 14 | 3 | 13 | 1 | 23 | 0 |
| *Syrna* | 7.94 | 14.48 | 40.87 | 70.46 | 41.62 | 38.47 | Kik | 250 | 15 | 2 | 11 | 2 | 0 | 13 | 2 | 2 | NA | NA | NA | NA |
| *Syros* | 83.78 | 127.92 | 7616.97 | 58.82 | 57.43 | 5.39 | Kik | 678 | 30 | 3 | 13 | 13 | 1 | 16 | 9 | 4 | 9 | 0 | 25 | 0 |
| *Thasos* | 383.75 | 472.28 | 472.28 | 8.00 | 0.00 | 0.00 | NAe | 1275 | 15 | 0 | 1 | 9 | 5 | 1 | 15 | 0 | NA | NA | 85 | 0 |
| *Thira* | 75.74 | 123.10 | 252.44 | 169.92 | 130.77 | 120.33 | Kik | 559 | 22 | 4 | 10 | 8 | 0 | 14 | 2 | 2 | 7 | 0 | NA | NA |
| *Tilos* | 61.83 | 99.55 | 134.76 | 21.02 | 20.40 | 20.62 | EAe | 516 | 16 | 3 | 9 | 4 | 0 | 12 | 9 | 3 | 11 | 0 | 20 | 0 |
| *Tinos* | 233.43 | 734.17 | 7616.97 | 50.93 | 10.77 | 5.39 | Kik | 822 | 34 | 5 | 11 | 17 | 1 | 16 | 12 | 4 | 8 | 0 | 29 | 0 |
| *Ydra* | 49.75 | 82.36 | 82.36 | 2.83 | 2.83 | 0.00 | Pe | 401 | 20 | 2 | 2 | 15 | 1 | 4 | 5 | 0 | NA | NA | NA | NA |

Table A 1.2

Distribution of island type at Median and Late Glacial Maximum sea levels (MSL and LGM, respectively), including the islands of both the angiosperms and centipedes datasets (n = 84). MSL land-bridge islands include Chios, Skiathos, Thasos, Pserimos and Kos. LGM land-bridge islands include: Agathonisi, Agios Efstratios, Aigina, Alonnisos, Ikaria, Kalymnos, Kea, Leipsoi, Lesvos, Limnos, Patmos, Samothraki, Skopelos, Ydra, Chios, Kos, Pserimos, Skiathos, Thasos, Kastellorizo, Stroggyli, Ro.

|  | Sea level | |
| --- | --- | --- |
|  | MSL | LGM |
| Land-bridge | 5 | 22 |
| True island | 79 | 62 |

Appendix 2:

Species-area relationships for selected chorotypes of angiosperms, butterflies, centipedes and reptiles on land-bridge islands and true islands. In blue dots the data points and regression lines for land-bridge islands, in green triangles the true islands. NNE stands for ‘native non endemics’; EAll for ‘all endemics’; AE for ‘Aegean endemics’; SIEs for ‘single island endemics’; GE for ‘Greek endemics’ and; MIEs for ‘multiple island endemics’. Goodness of the fit values are adjusted-R^2^ (poor fit can result in negative values). MSL island type was used for the NNE angiosperm, NNE butterflies and NNE reptiles ISARs (see GLMMs selection) whereas LGM island type was used for all remaining taxa and chorotypes.
