# Supplementary figures and images for: Past connections with the mainland structure patterns of insular species richness in a continental‐shelf archipelago (Aegean Sea, Greece)
Source: Ecol Evol. 2021 Mar 29;11(10):5441–58. doi: 10.1002/ece3.7438 (PMC8131802; doi:10.1002/ece3.7438)

# AE angiosperms

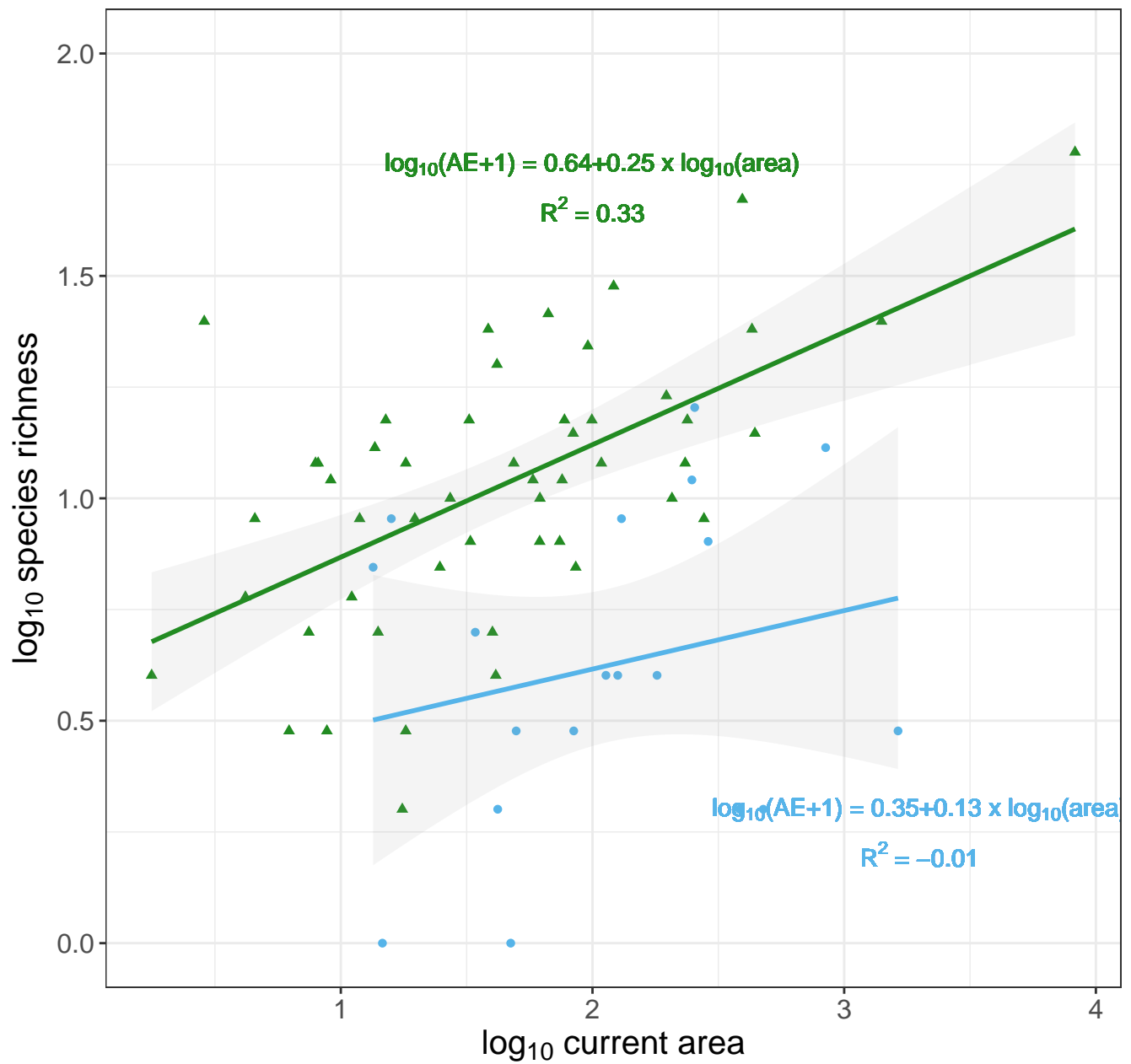

Supplement: Supplementary file 2 — Supplementary Material [file ECE3-11-5441-s003.pdf]

# EAll angiosperms

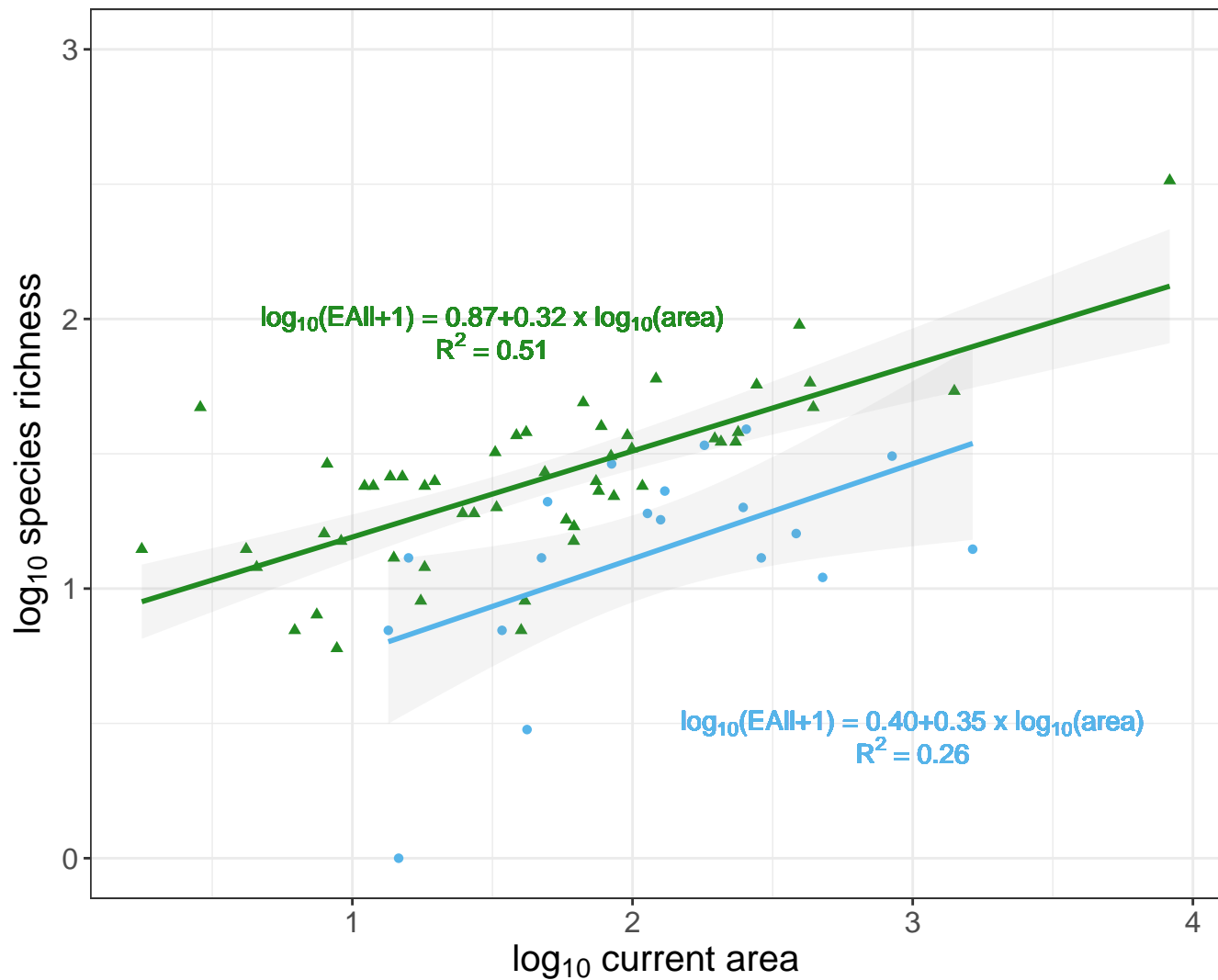

Supplement: Supplementary file 3 — Supplementary Material [file ECE3-11-5441-s002.pdf]

# EAll reptiles

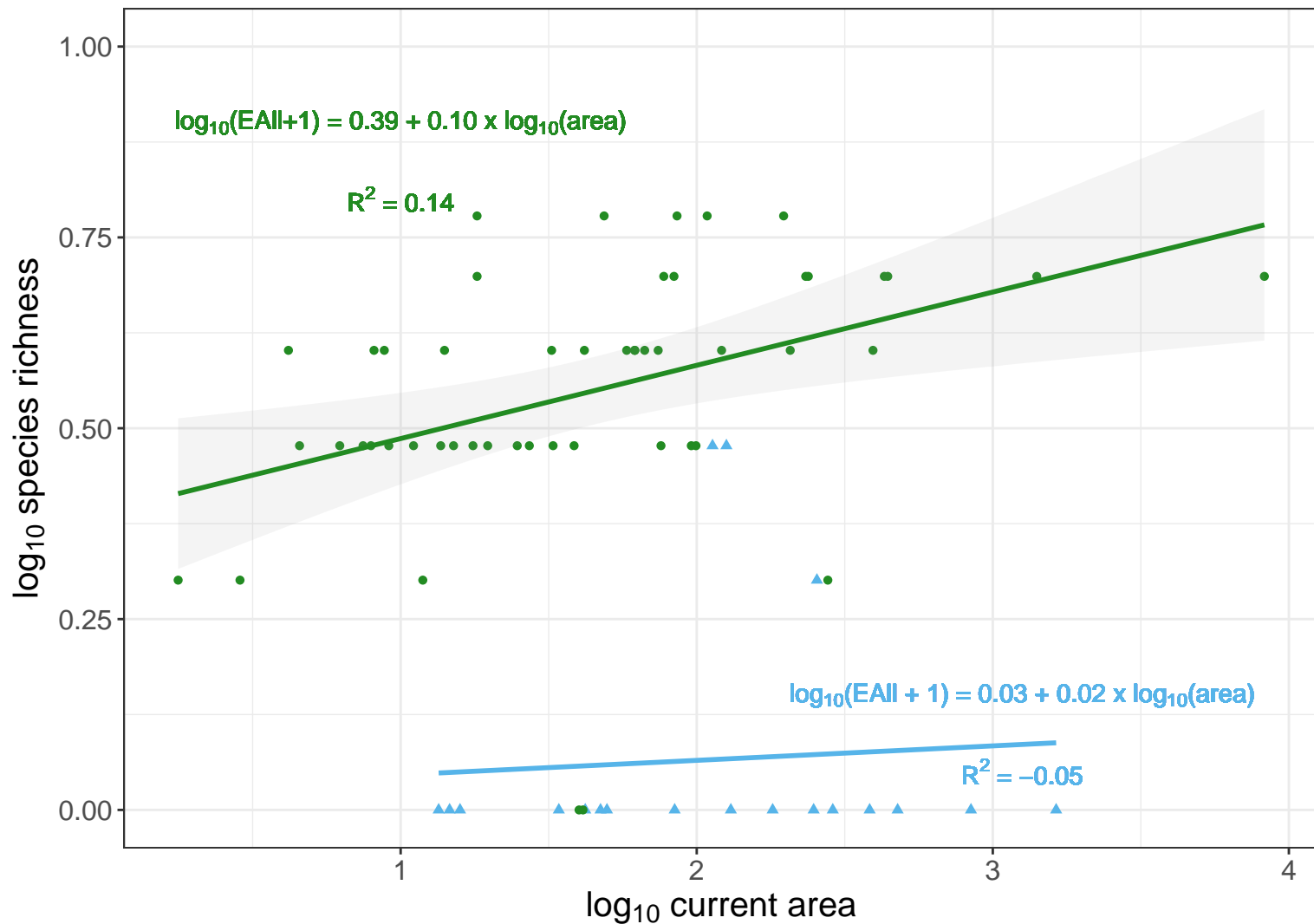

Supplement: Supplementary file 4 — Supplementary Material [file ECE3-11-5441-s001.pdf]

# GE angiosperms

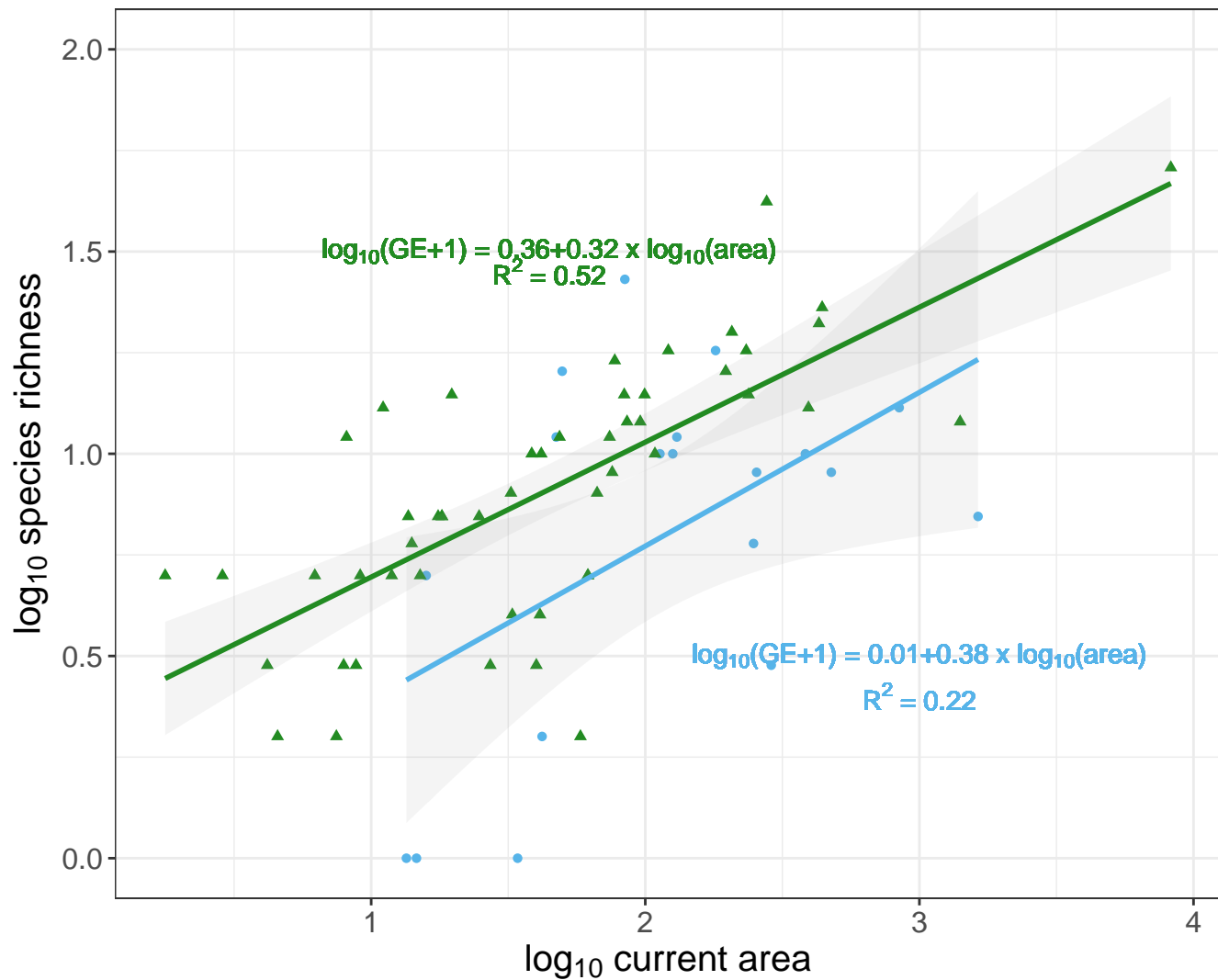

Supplement: Supplementary file 5 — Supplementary Material [file ECE3-11-5441-s004.pdf]

# MIEs angiosperms

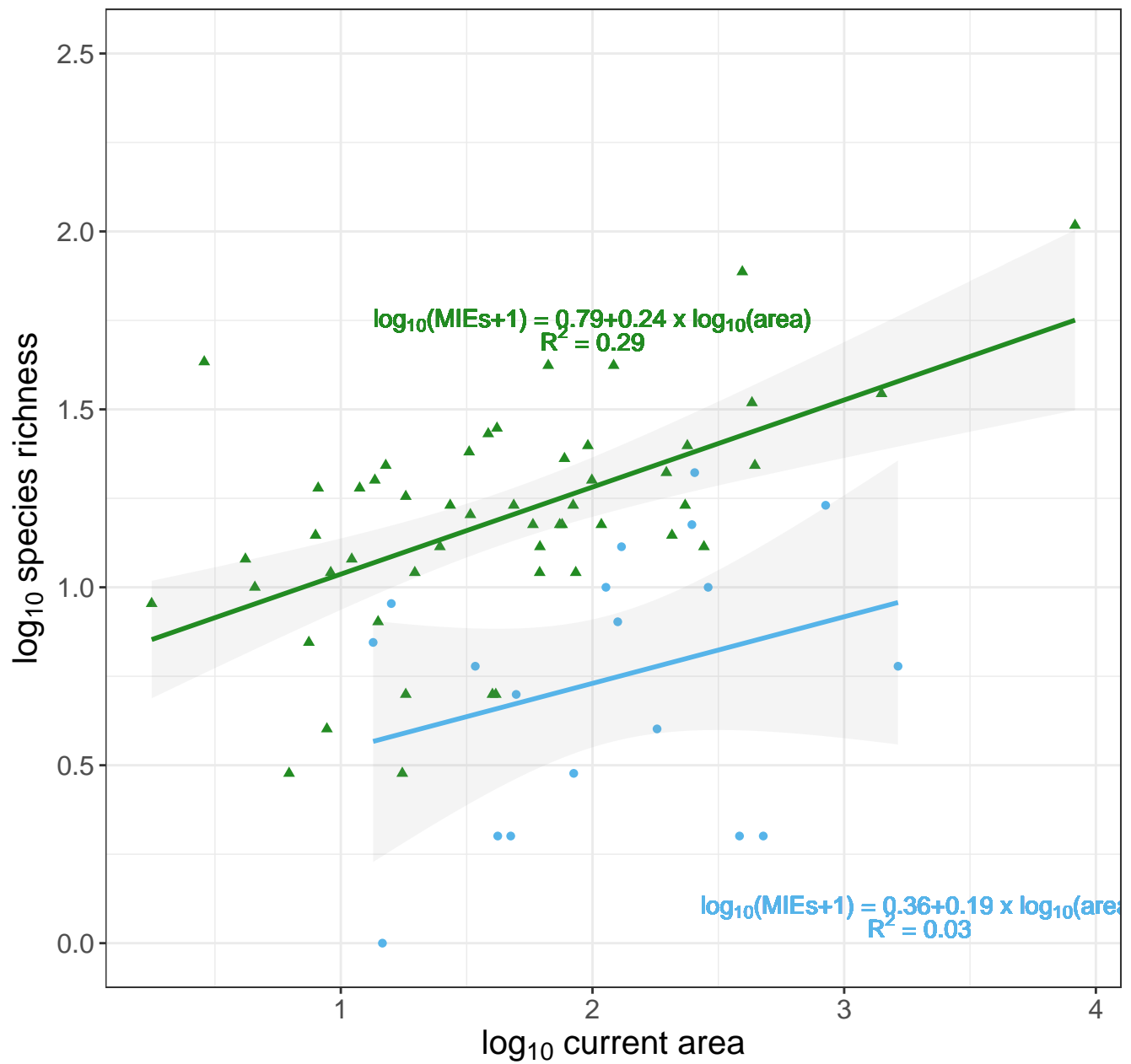

Supplement: Supplementary file 6 — Supplementary Material [file ECE3-11-5441-s010.pdf]

# NNE angiosperm

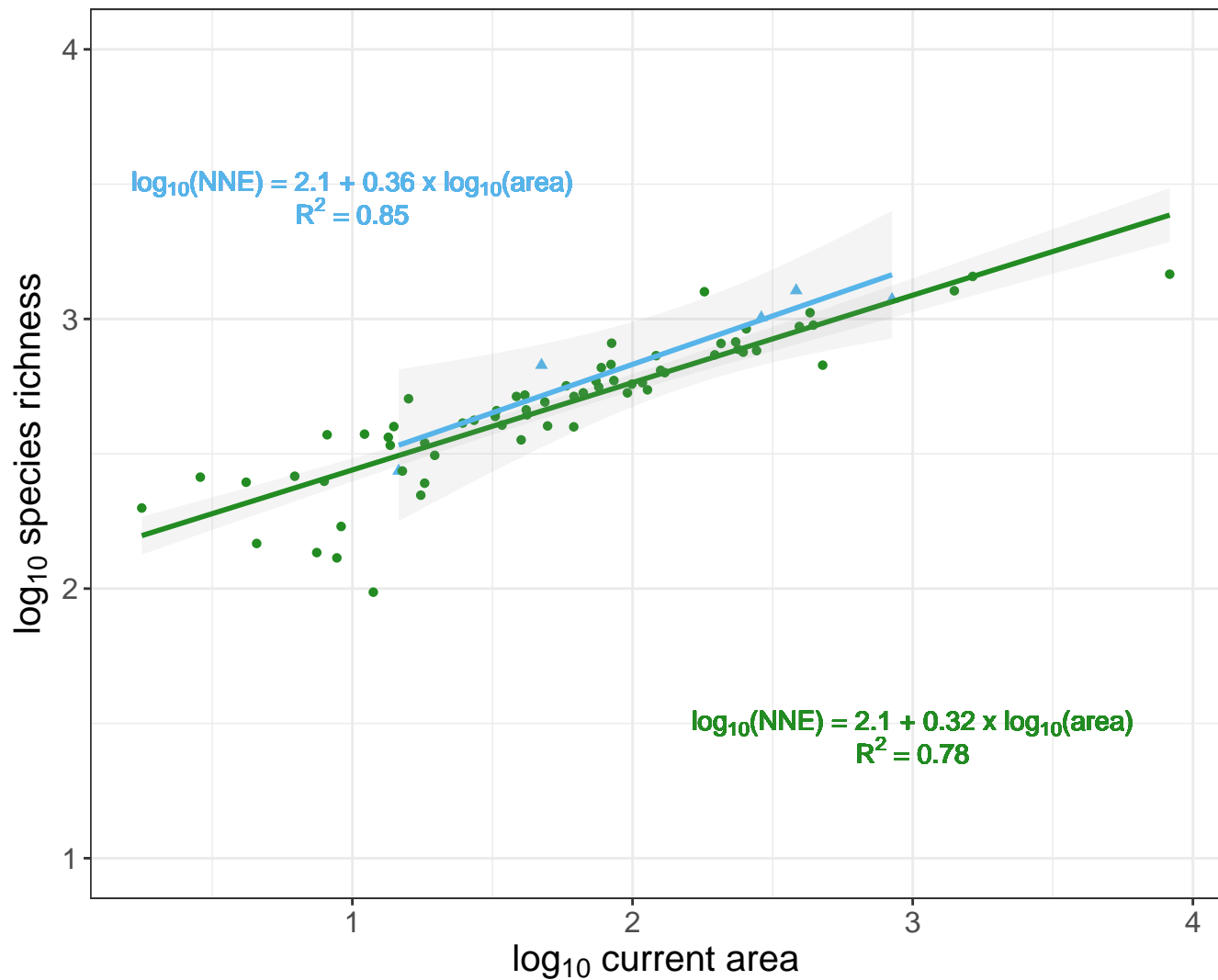

Supplement: Supplementary file 7 — Supplementary Material [file ECE3-11-5441-s011.pdf]

# NNE butterflies

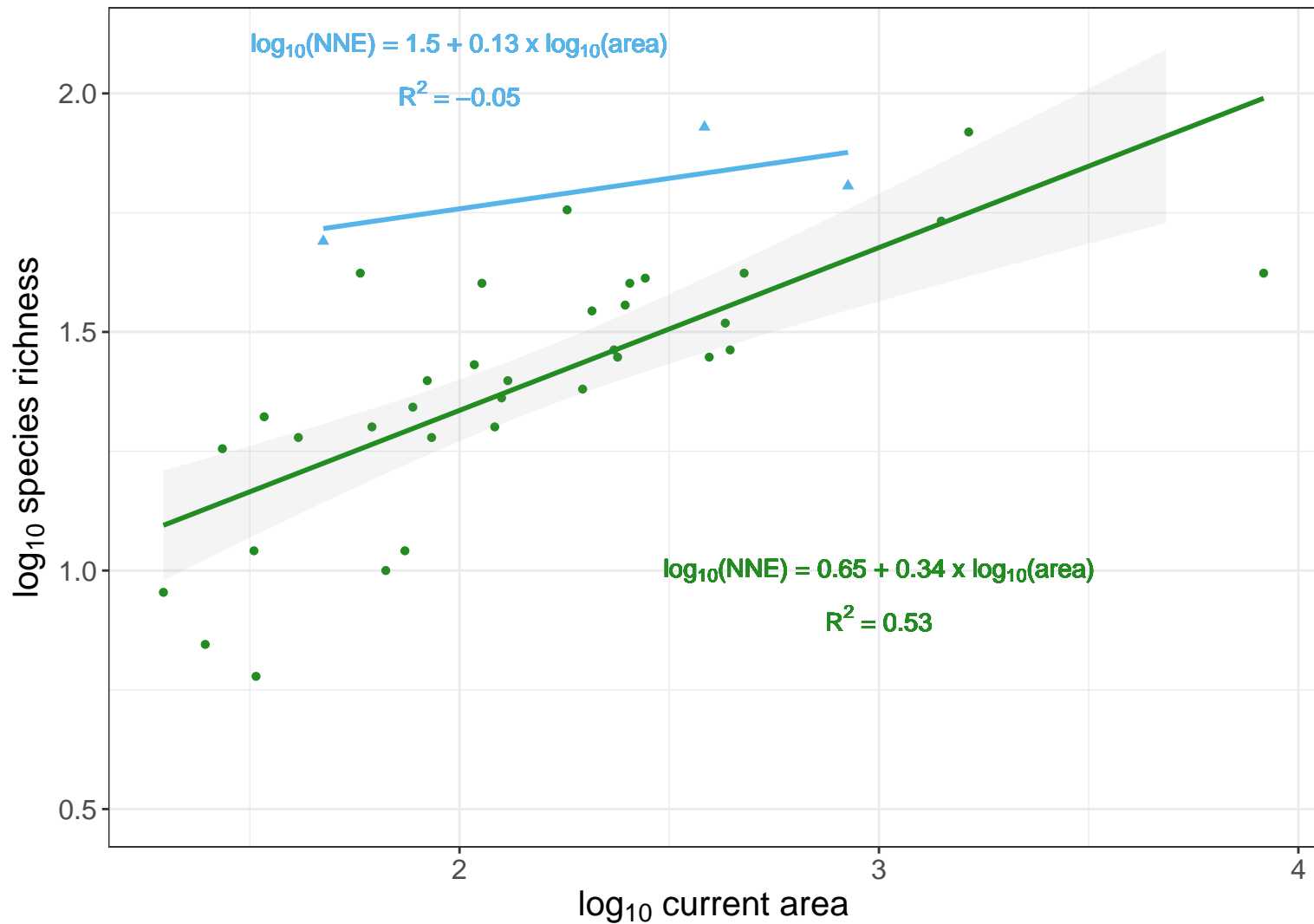

Supplement: Supplementary file 8 — Supplementary Material [file ECE3-11-5441-s005.pdf]

# NNE centipedes

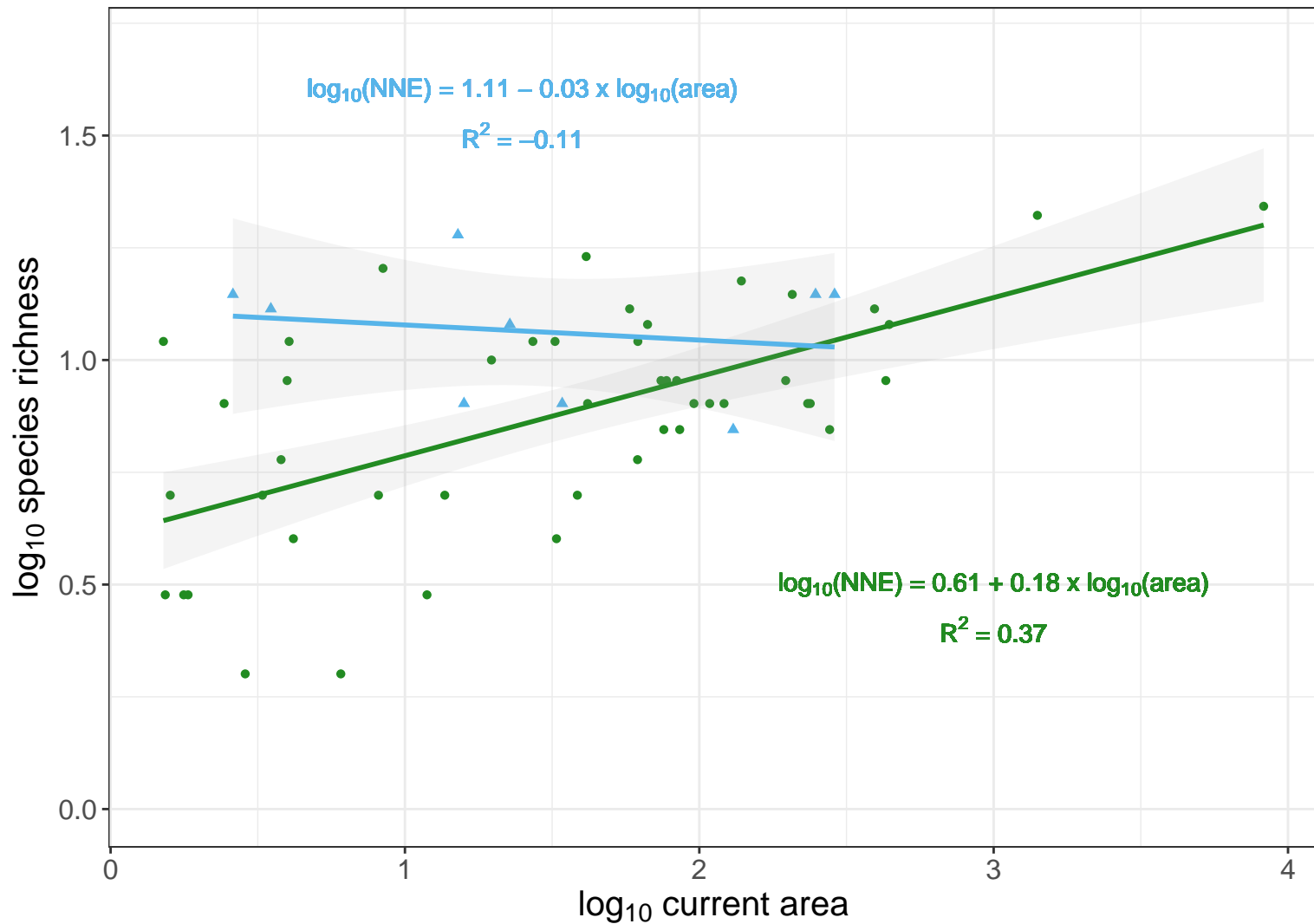

Supplement: Supplementary file 9 — Supplementary Material [file ECE3-11-5441-s009.pdf]

# NNE reptiles

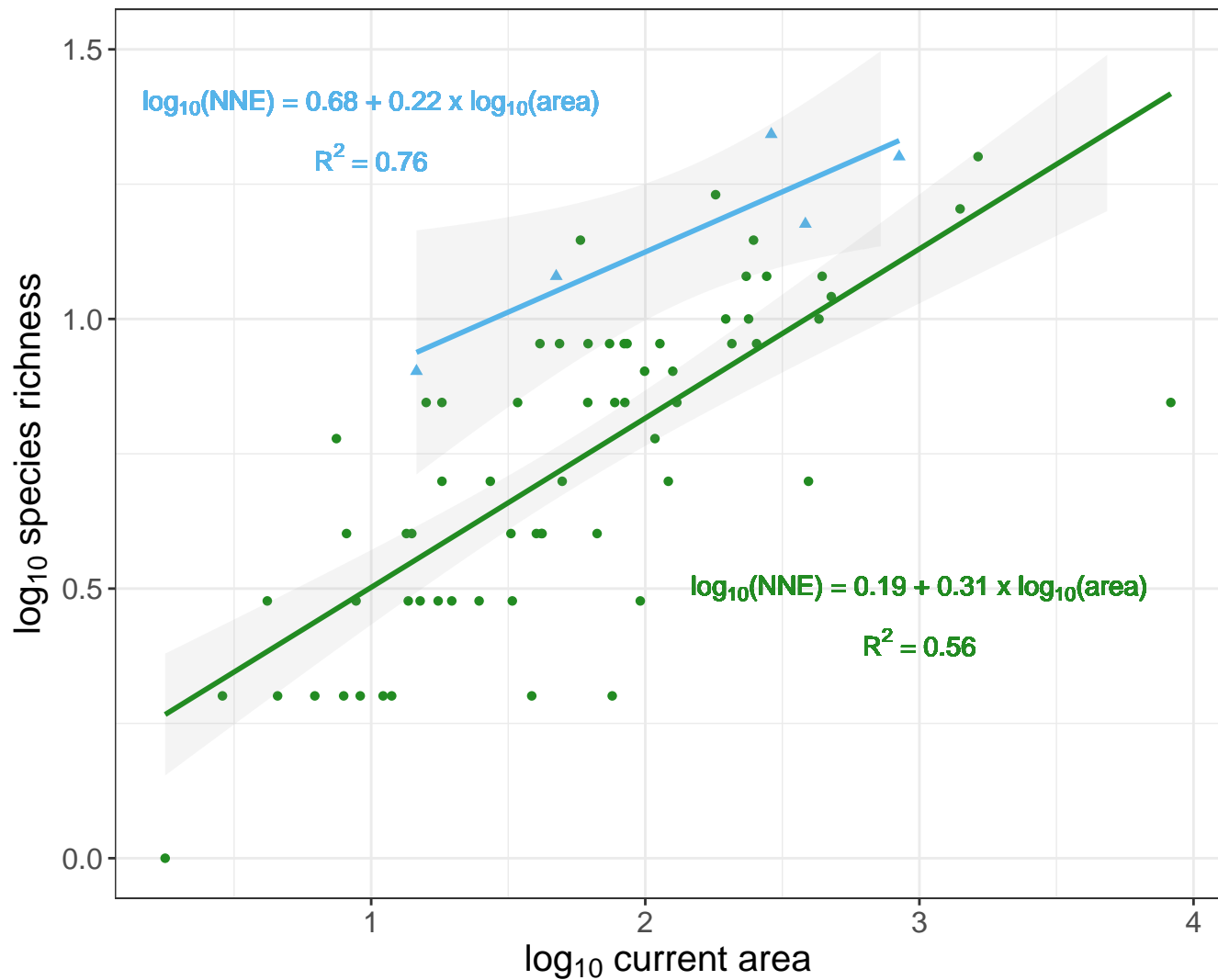

Supplement: Supplementary file 10 — Supplementary Material [file ECE3-11-5441-s007.pdf]

# SIEs angiosperms

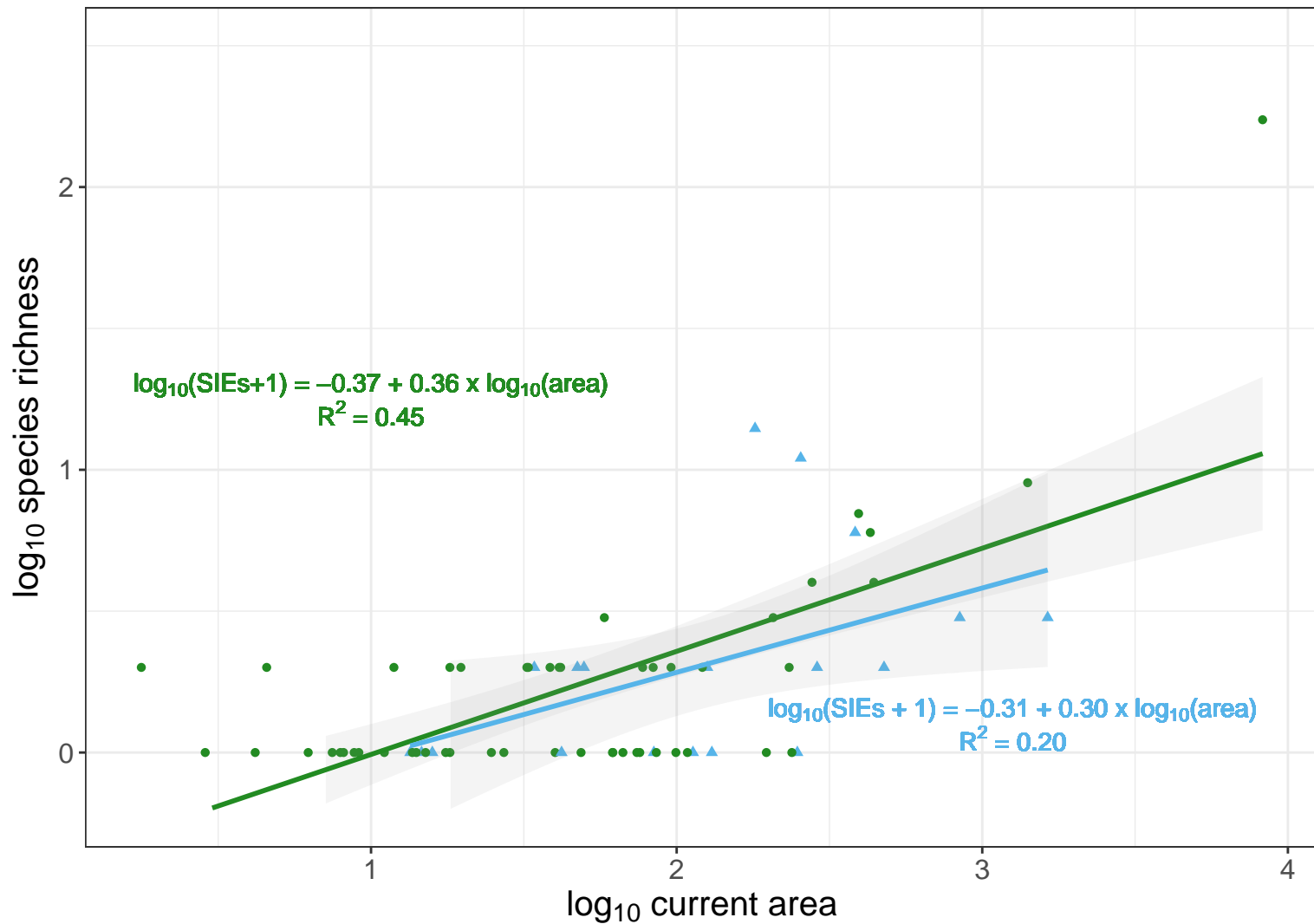

Supplement: Supplementary file 11 — Supplementary Material [file ECE3-11-5441-s006.pdf]
